# Identification of new binding proteins of focal adhesion kinase using immunoprecipitation and mass spectrometry

**DOI:** 10.1038/s41598-019-49145-6

**Published:** 2019-09-09

**Authors:** Binh Thanh Nguyen, Jae-Chul Pyun, Sang-Guk Lee, Min-Jung Kang

**Affiliations:** 1 Molecular Recognition Research Center, Korea Institute of Science and Technology (KIST), Seoul, 02792 South Korea; 2 Division of Bio-Medical Science and Technology (Biological Chemistry), Korea University of Science and Technology (UST), Daejeon, 34113 South Korea; 3 Department of Materials and Sciences, Yonsei University, Seoul, 120-749 South Korea; 4 Department of Laboratory Medicine, Severance Hospital, Seoul, 120-752 South Korea; 5 Yonsei University College of Medicine, Seoul, 120-752 South Korea

**Keywords:** Targeted therapies, Protein-protein interaction networks

## Abstract

Focal adhesion kinase (FAK) is a 125 kDa protein recruited as a participant in focal adhesion dynamics and serves as a signaling scaffold for the assembly and subsequent maturation of focal contact. Identification of new FAK binding proteins could reveal potential signaling targets and contribute to further development of therapeutic drugs in the treatment of colon cancer. Here, we applied a functional proteomic strategy to identify proteins that interact with FAK in human colon cancer cell line HCT-116. Proteins were targeted by coimmunoprecipitation with an anti-FAK antibody and resolved on 1D-SDS-PAGE. The gel was excised, reduced, alkylated, and trypsin digested. Tryptic peptides were separated by nano-LC-MS/MS by an LTQ-Orbitrap-Velos spectrometer. We identified 101 proteins in the immunocomplex under epithelial growth factor (EGF) stimulation. Three proteins, zyxin, nesprin-1, and desmoplakin, were discovered and validated using reciprocal immunoprecipitation and Western blot analysis. Then, we sought to study the biological relevance of these proteins by siRNA transfection of HCT-116 cells. According to the results, zyxin might play a central role as an upstream regulator to mediate critical cancer-related signaling pathways. Zyxin and nesprin-1 depletion significantly impaired cell migration and invasion capabilities. Additionally, we performed ELISA assays on serum samples from patients with colon cancer instead of cell models to quantify the protein levels of zyxin and nesprin-1. Our results suggested that zyxin and nesprin-1 are not only promising therapeutic targets but also potential diagnostic biomarkers for colon cancer.

## Introduction

Colon cancer is one of the most commonly diagnosed cancers and the leading cause of cancer death, with an estimated 1,096,601 cases and 551,269 deaths in 2018 according to Global Cancer Statistics^1,2^. Although colon cancer can be cured at early stages, the symptoms are frequently neglected because the signs are the same as those of common abdominal noncancerous conditions, such as hemorrhoids and irritable bowel syndrome^3,4^. Numerous patients with colon cancer do not express any symptoms until metastasis occurs, thus leading to an extremely low survival rate and ineffective treatment. Although chemotherapy has served as the backbone of cancer treatment, its cytotoxicity destroys cancer cells as well as surrounding healthy tissues, resulting in severe side effects, including hair loss, nausea, infections, and immune system destruction. In some instances, these effects may recur months or years post-treatment. Currently, several drugs are approved by the U.S. Food and Drug Administration (FDA) to treat colon cancer in the U.S^5^. However, researchers have been seeking alternative strategies to replace or combine with traditional chemotherapy to enhance the efficacy of cancer treatment and to limit the nonspecific consequences and side effects of chemotherapy treatment. An emerging approach is targeted therapy; they involve targeting specific genes or proteins found in cancer cells, thus preventing cancer from growing and metastasizing. For colon cancer, conventional targeted therapies include epithelial growth factor receptor (EGFR) inhibitors (cetuximab and panitumumab)^6,7^, which slow down cancer growth, or vascular endothelial growth factor (VEGF) inhibitors (bevacizumab, ramucirumab, and Ziv-aflibercept)^6,8^, which suppress the angiogenesis process. Despite being cutting-edge cancer treatments, targeted therapies may face temporary setbacks as cancer cells tend to mutate to protect themselves from therapeutics. For example, 40% of colon cancer patients have the KRAS gene mutation, leading to the ineffectiveness of targeted therapeutics cetuximab and panitumumab^9^.

Accordingly, developing new therapeutic or diagnostic targets for colon cancer to improve patient quality of life is imperative.

Focal adhesion kinase (FAK) or protein tyrosine kinase 2 PTK2 is expressed ubiquitously in mammals and lower eukaryotic organisms^10,11^. The regulation of FAK has been reported to engage in several cellular activities, including cell growth, proliferation, differentiation, and apoptosis. FAK plays a critical role in tumor progression and cancer metastasis via its regulation of both cancer cells and their activities, such as migration, invasion, and epithelial-mesenchymal transition (EMT)^12–14^. The vital regulatory role of FAK in these diverse biological processes makes FAK an important drug target in the diagnosis and treatment of various diseases^15,16^. Since the discovery of FAK, a large number of studies have focused on its therapeutic use in various cancers, including ovarian, lung, kidney, brain, pancreatic, breast and prostate cancers^17–22^. For example, VS-4718 is an orally bioavailable FAK inhibitor with potential antineoplastic activity. Upon administration, VS-4718 inhibits FAK, blocks fibronectin-stimulated FAK autophosphorylation of Tyr397, and may prevent the integrin-mediated activation of numerous downstream signal transduction cascades, including ERK, JNK/MAPK and PI3K/AKT. This treatment results in a decline in cancer stem cells (CSCs) and suppresses tumor cell migration, proliferation, and survival^22^.

According to several studies, overexpression of FAK is correlated with metastatic colon cancer^23–25^. However, FAK binding partners and their interaction dynamics that regulate the physiological and pathological processes in colorectal cancer have not yet been fully elucidated. The versatile structure of FAK increases the number of additional binding partners that interact with FAK via indirect or secondary interactions. Therefore, identification of the interactome of FAK is necessary to understand the regulation of this oncogene and target new binding partners as potential therapeutics for colon cancer.

Mass spectrometry (MS) has recently become an evolving tool to identify proteins in biological samples, placing MS at the top leading technologies to investigate protein-protein interactions^26–28^. Although the yeast two-hybrid screening method is widely utilized to study protein-protein interactions, it commonly produces false-positive results, such as detecting protein pairs that can interact but do not necessarily associate *in vivo*^29–31^. MS-based methods can be combined with a wide range of protein purification and protein capture strategies, such as immunoprecipitation (IP), thus allowing rapid and reliable identification of components^32,33^.

In this work, we studied the interaction network of FAK in HCT-116 colon cancer cells by applying the IP method followed by LC-MS/MS peptide sequencing. We have identified among FAK interactors; zyxin, a zinc-binding phosphoprotein located along the actin cytoskeleton which is known to involve in cell motility^34^; nesprin-1, a protein shuttles between actin filaments and the nucleus is crucial for nuclear positioning and anchorage^35^; and desmoplakin, a desmosomal protein which maintains structural integrity at cell-cell interfaces^36^. We confirmed and validated new candidates by reciprocal IP and Western blot analysis. Furthermore, their functions in migration and invasion were investigated, which are initially involved in tumor progression and chaperone activities. In addition, we performed ELISA assays to quantify the protein levels in sera from patients with colon cancer.

## Results

### Identification of FAK-interacting proteins in human colon cancer HCT-116 cells

**Figure 1:**
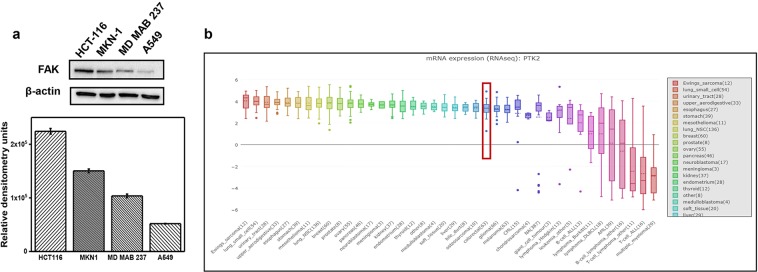
(**a**) Western blot analysis of FAK expression levels in different cancer cell lines. HCT-116: colon cancer; MKN-1: gastric cancer; MD MAB237: breast cancer; A549: lung cancer. (**b**) mRNA expression (RNAseq) of PTK2, encoding the FAK protein gene among cancers. The family of colorectal cancer tissues demonstrated a high level of FAK. Data from Cancer Cell Line Encyclopedia (CCLE).

FAK protein is expressed in various cancers, including ovarian, cervical, kidney, prostate, brain, breast, and skin cancer. Our Western blot results showed that FAK protein levels were higher in colon cancer cell line HCT-116 than in gastric cancer cell line MKN-1, breast cancer cell line MD MAB237 and lung cancer cell line A549 (Fig. 1a). In addition, mRNA expression (RNAseq) of the PTK2 gene encoding the FAK protein provided by the Cancer Cell Line Encyclopedia database supports strong evidence of FAK overexpression in colorectal cancer (Fig. 1b).

To study the interaction network of FAK, we carried out IP of 1.5 mg total cell lysates extracted from HCT-116 cells using 10 µl anti-FAK polyclonal antibody conjugated with agarose beads. Rabbit serum with the same concentration of IgG as that of the antibody served as a negative control. To reduce nonspecific binding proteins, we precleared cell lysates with the same material as that used in IP, and solid beads were preblocked before incubation with the matrix.

**Figure 2:**
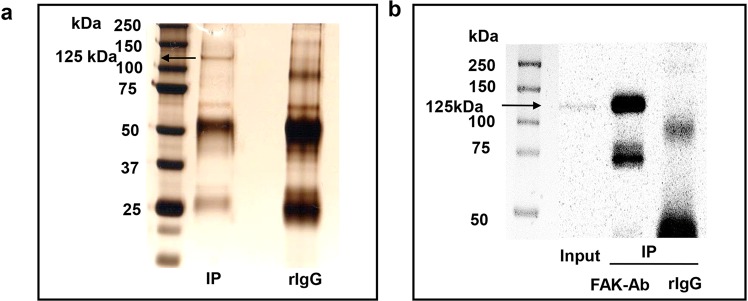
Immunoprecipitation of FAK. Proteins immunoprecipitated from total cell lysate from the HCT-116 cell line using a polyclonal anti-FAK antibody or rabbit IgG as a negative control. Eluents were electrophoresed on 10% SDS-PAGE. (**a**) Silver staining of one gel demonstrated a 125 kDa band corresponding to FAK that was present in the anti-FAK immunoprecipitate HCT-116 lysates but not in the IgG control. (**b**) After SDS-PAGE, the gel was transferred to a nitrocellulose membrane and immunoblotted using FAK antibody. A band of 125 kDa corresponding to FAK was identified in the total cell lysate or anti-FAK IP but not in the IgG control.

A 125 kDa band corresponding to FAK was identified in the FAK pulldown lane or input lane but not in the IgG control lane on a silver stained gel (Fig. 2a) and Western blot (Fig. 2b). These data demonstrate that FAK can be efficiently and specifically immunoprecipitated from the cell extract.

Immunoprecipitated proteins were gel excised, trypsin digested and analyzed by nano liquid chromatography tandem mass spectrometry (Gel-nanoLC-MS/MS). We identified 228 known database entries, and 101 proteins were screened reproducibly using strict filtration described in the experimental procedure. These candidate proteins were found in at least two out of three regular IPs or IP under epithelial growth factor (EGF) stimulation. Fifteen proteins were found exclusively by regular IP (Supplementary Table S1), 18 were found solely by IP with EGF treatment, and 83 mutual proteins were found in both the regular IP and stimulated IP (Supplementary Fig. S1). As expected, FAK was identified among precipitated proteins with a sequence coverage of more than 40%. The MS full scan and MS/MS scan for peptides matching FAK are shown in Supplementary Fig. S2 and Supplementary Table S2.

**Figure 3:**
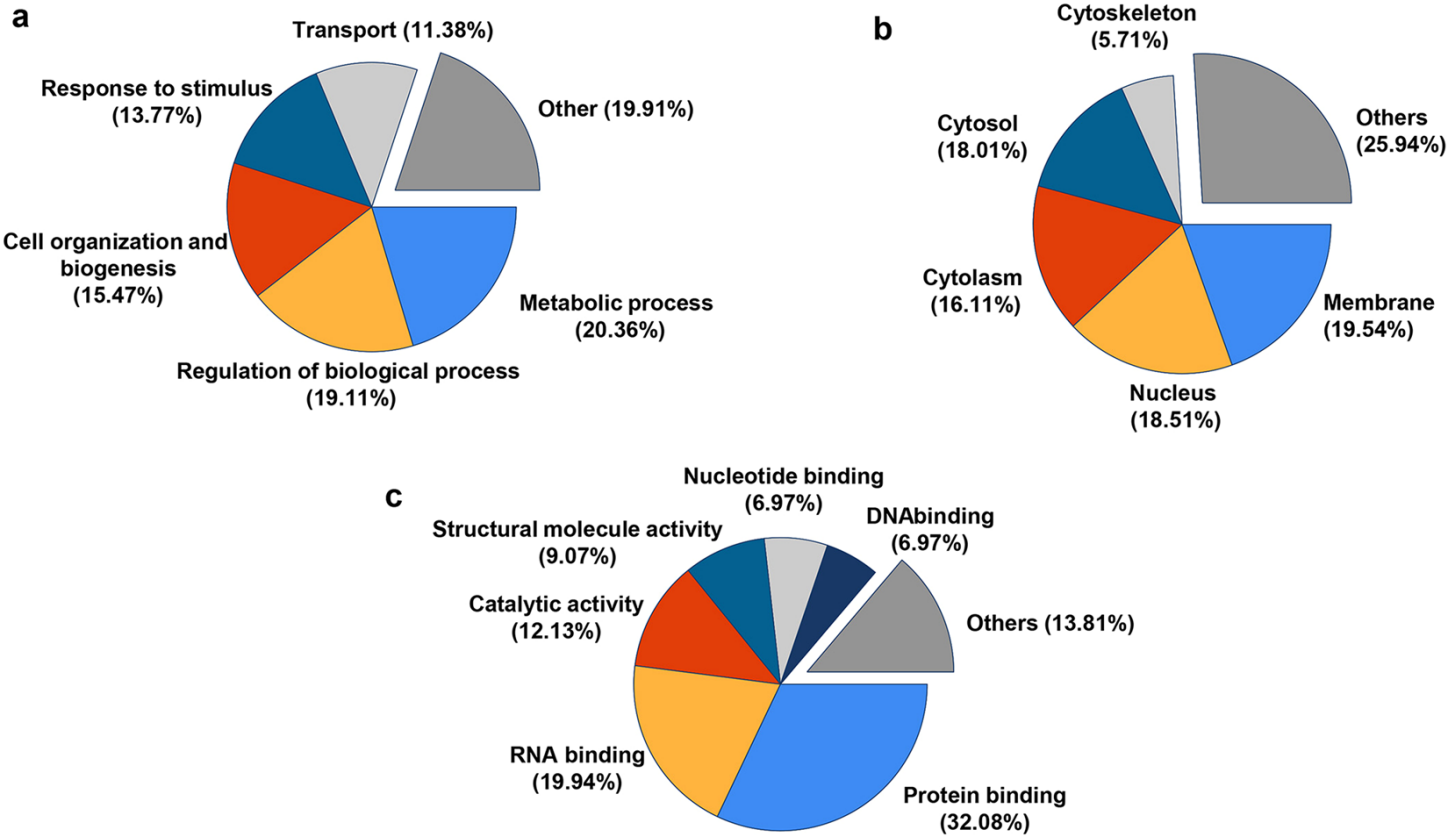
Biological relevance characterization of identified proteins using IP-1Dgel-LC/MSMS analysis. Pie-chart representation of (**a**) Functional process. (**b**) Cellular location. (**c**) Molecular function.

To distinguish bona fide proteins that specifically interact with FAK and not those associated with IgG in a nonspecific manner, we eliminated proteins that were commonly found between the IgG control IP lane and FAK pulldown lane together with contaminants such as the keratin family and applied the stringent inclusion criteria described in the Materials and Methods. FAK was found along with previously reported binding proteins PAX^37^, GRB7^38^, MAP4^39^, ELAV1^40^, PPP1CB^41^, ACTB^42^, and HSP90AB1^43^. A complete list of identified peptides is provided in Tables 1 and 2. The pulled-down proteins are largely involved in structural molecule binding, followed by DNA and RNA functional binding. Metal ion binding proteins showed no interaction with FAK. The most significant biological process was related to cell organization. Numerous immunoprecipitated proteins were anchored on cytoskeleton filaments or membranes, whereas proteins in mitochondria were absent in the pulldown mixture. These data indicate that FAK plays a central role in diverse cellular functions related to widespread biological processes (Fig. 3 and Supplementary Fig. S4).

**Table 1:** Identification of FAK interacting proteins by IP-1D gel-LC/MSMS.

Accession	Description	X_corr_ (Top charge)	Score	Abundance ratio
Q05397	**Focal adhesion kinase 1**	4.44 (+3)	210.63	3.64
P17844	probable ATP-dependent RNA helicase DDX5	4.67 (+2)	131.15	6.037
O00571	ATP-dependent RNA helicase DDX3X	4.88 (+3)	108.31	2.595
Q9BVP2	Guanine nucleotide-binding protein-like 3	4.68 (+3)	104.89	5.905
P15924-1	Desmoplakin	4.05 (+3)	68.1	4.72
P46087-4	Isoform 4 of Probable 28 S rRNA (cytosine(4447)-C(5))-methyltransferase	4.62 (+2)	65.1	4.632
P12236	ADP/ATP translocase 3	3.37 (+2)	59.43	0.939
Q15050	Ribosome biogenesis regulatory protein homolog	3.89 (+3)	40.46	1.112
P43243	Matrin-3	3.39 (+3)	39.76	2.579
Q15942	Zyxin	4.47 (+2)	38.4	1.34
Q13283	Ras GTPase-activating protein-binding protein 1	3.97 (+3)	37.89	8.771
P60709	**Actin, cytoplasmic 1**	3.40 (+2)	33.51	6.08
P22087	rRNA 2’-O-methyltransferase fibrillarin	3.54 (+2)	31.45	0.665
Q12797	Aspartyl/Asparaginyl beta-hydroxylase	3.64 (+2)	30.64	3.523
Q9Y4P3	Transducin beta-like protein 2	3.47 (+2)	30.26	0.814
P52292	Importin subunit alpha 1	4.25 (+3)	30.53	6.58
P54136-1	arginine–tRNA ligase, cytoplasmic	4.18 (+3)	28.97	4.158
Q9UMS4	Pre-mRNA-processing factor 19	3.35 (+3)	28.07	7.427
P23246-1	splicing factor, proline- and glutamine-rich	3.79 (+3)	27.97	9.459
Q6NVV1	Putative 60 S ribosomal protein L13a protein RPL13AP3	3.10 (+2)	27.59	0.583
O75533-1	splicing factor 3B subunit 1	4.50 (+3)	26.28	1.973
P20700	Lamin-B1	3.59 (+2)	26.23	2.929
Q96E39	RNA binding motif protein, X-linked-like-1	3.80 (+2)	24.58	0.679
Q06787	synaptic functional regulator FMR1	3.54 (+3)	22.12	3.614
O00541-1	Pescadillo homolog	2.85 (+2)	21.59	6.783
Q14444-1	Caprin-1	3.90 (+3)	21.57	5.886
P49023	**Paxillin**	4.04 (+2)	21.34	2.668
Q9NVV4-2	Isoform 2 of Poly(A) RNA polymerase, mitochondrial	3.50 (+2)	20.85	4.826
P38646	Stress-70 protein, mitochondrial	2.38 (+2)	19.55	0.666
Q03252	Lamin-B2	2.34 (+2)	17.57	4.195
Q16630-2	Cleavage and polyadenylation specificity factor subunit 6	3.74 (+2)	17.15	11.955
Q07666	KH domain-containing, RNA-binding, signal transduction-associated protein 1	2.46 (+2)	15.71	6.672
Q96EY1-1	DnaJ homolog subfamily A member 3, mitochondrial	3.54 (+3)	14.87	1.351
P51991-1	Heterogeneous nuclear ribonucleoprotein A3	2.76 (+2)	13.9	0.804
Q9BQ70	Transcription factor 25	3.78 (+3)	12.63	3.148
P07355	Annexin A2	3.60 (+2)	12.41	0.477
Q9H6F5	Coiled-coil domain-containing protein 86	3.65 (+3)	12.39	7.331
Q5C9Z4	nucleolar MIF4G domain-containing protein 1	3.78 (+2)	12.24	1.68
Q5JTH9-1	RRP12-like protein	3.36 (+3)	12.19	0.755
Q9Y446	Plakophilin-3	2.31 (+2)	12.12	2.158
Q9NZI8	Insulin-like growth factor 2 mRNA-binding protein 1	3.65 (+3)	11.65	4.243
P22626	heterogeneous nuclear ribonucleoproteins A2/B1	2.74 (+2)	11.46	1.036
Q99623	Prohibitin-2	2.47 (+2)	10.75	0.325
P62140	**Serine/threonine-protein phosphatase PP1-beta catalytic subunit**	3.64 (+2)	10.36	1.32
Q9H7E9-1	UPF0488 protein C8orf33	3.91 (+2)	10.28	0.906
Q9NQZ2	Something about silencing protein 10	3.08 (+3)	10.08	3.312
O15371	Eukaryotic translation initiation factor 3 subunit D	3.04 (+3)	9.96	5.524
Q96AG4	Leucine-rich repeat-containing protein 59	2.84 (+2)	9.76	2.725
O43795	Unconventional myosin-Ib	2.62 (+2)	9.33	27.914
P52597	Heterogeneous nuclear ribonucleoprotein F	2.42 (+2)	9.13	2.622
Q7L0Y3	Mitochondrial ribonuclease P protein 1	3.43 (+3)	9.01	0.81
A0A0C4DH55	immunoglobulin kappa variable 3D-7	3.49 (+3)	8.65	1.785
P21796	voltage-dependent anion-selective channel protein 1	2.93 (+2)	8.62	3.353
P27816-1	**Microtubule-associated protein 4**	2.44 (+2)	8.62	15.012
Q15046-2	Isoform Mitochondrial of Lysine–tRNA ligase	2.75 (+2)	8.52	2.74
P36542-1	ATP synthase subunit gamma, mitochondrial	3.20 (+2)	8.42	0.97
P04843	Dolichyl-diphosphooligosaccharide–protein glycosyltransferase subunit 1	2.55 (+2)	8.28	3.656
O43663-1	Protein regulator of cytokinesis 1	3.47 (+3)	8.06	5.937
P68104	Elongation factor 1-alpha 1	3.20 (+3)	7.22	2.923
Q15365	Poly(RC)-binding protein 1	3.39 (+2)	6.99	1.148
Q8N0V3-1	Putative ribosome-binding factor A, mitochondrial	2.59 (+2)	5.56	1.918
Q14451	**Growth factor receptor-bound protein 7**	2.95 (+2)	5.28	3.678
Q8WU90	Zinc finger CCCH domain-containing protein 15	2.42 (+2)	5.22	5.429
P62805	**histone H4**	3.37 (+3)	5.2	0.139
Q96GM8	Target of EGR1 protein 1	3.64 (+3)	4.73	4.628
P45880-1	Isoform 1 of Voltage-dependent anion-selective channel protein 2	3.22 (+3)	4.69	0.136
Q9UJZ1	Stomatin-like protein 2, mitochondrial	2.27 (+2)	4.45	0.01
O00159-2	Isoform 2 of Unconventional myosin-Ic	3.93 (+3)	3.93	100
Q9UHB6-3	Isoform 3 of LIM domain and actin-binding protein 1	3.82 (+3)	3.82	100
P30050-1	60 S ribosomal protein L12	3.50 (+2)	3.5	0.378
Q5D862	Filaggrin-2	3.38 (+2)	3.38	0.01
P14923	Junction plakoglobin	3.29 (+3)	3.29	0.976
Q15717	**ELAV-like protein 1**	3.22 (+2)	3.27	0.784
P62979	Ubiquitin-40S ribosomal protein S27a	3.20 (+2)	3.25	0.79
O00515	Ladinin-1	2.81 (+2)	3.24	1.01
P62899-2	Isoform 2 of 60 S ribosomal protein L31	2.61 (+2)	3.22	0.244
Q9NQT5	exosome complex component RRP40	2.51 (+2)	3.21	0.01
Q9HCM4-4	Isoform 4 of Band 4.1-like protein 5	2.29 (+2)	3.2	4.986
Q4G0J3-3	Isoform 3 of La-related protein 7	2.94 (+2)	3.14	2.627
Q99496	E3 ubiquitin-protein ligase RING2	2.61 (+3)	3.04	1.517
P08238	**Heat shock protein HSP 90-beta**	3.97 (+2)	2.97	100
O15213	WD repeat-containing protein 46	2.34 (+2)	2.53	100
P14778	Interleukin-1 receptor type 1	2.67 (+2)	2.51	0.281

**Table 2:** Proteins identified exclusively by IP-EGF stimulation.

Accession	Description	Xcorr (Top charge)	Score	Abundance ratio
O43707	**Alpha-actinin-4**	1.94 (+1)	58.3	100
P12814-4	**Isoform 4 of Alpha-actinin-1**	2.43 (+2)	38.89	100
Q01082-1	Spectrin beta chain, non-erythrocytic 1	3.21 (+3)	19.61	100
O75369-8	Isoform 8 of Filamin-B	2.32 (+3)	15.26	100
P21333	Filamin-A	2.78 (+2)	11.33	100
Q07157	Tight junction protein ZO-1	3.48 (+3)	7.53	100
O75044	SLIT-ROBO Rho GTPase-activating protein 2	2.67 (+2)	5.36	100
Q14315	Filamin-C	3.75 (+3)	4.87	100
Q16643-3	Isoform 3 of Drebrin	3.21 (+3)	4.7	100
B1AK53-1	espin	3.01 (+3)	3.19	100
P13797	Plastin-3	2.37 (+2)	2.95	100
Q8IYE0-1	Coiled-coil domain-containing protein 146	3.21 (+3)	2.64	100
P14902	Indoleamine 2,3-dioxygenase 1	3.43 (+3)	2.41	100
Q9BTV4	Transmembrane protein 43	3.60 (+3)	2.32	100
Q8IZH2-1	cingulin	3.97 (+3)	2.39	100
Q8IZH2-1	5′-3′ exoribonuclease 1	2.13 (+2)	2.16	100
O94832	Unconventional myosin-Id	2.82 (+2)	2	100
Q8NF91	Nesprin-1	3.45 (+2)	1.63	100

Bold entries indicate previously reported FAK interactions.

### FAK inhibition correlated with several binding proteins

To support the potential candidates for further validation, we conducted in-solution digestion using cell lysates extracted from HCT-116 cells treated with FAK inhibitors VS-6063 (Defactinib) and PF-573228 (Supplementary Fig. S3). Then, we calculated the fold change in proteins between the control and drug tests in terms of dose dependence. Several protein abundances significantly diminished upon the combination of two inhibitors. Among the previously identified proteins from IP, zyxin was consistently correlated with FAK inhibitors individually. Upon cotreatment, zyxin was completely suppressed (Table 3).

**Table 3:** Proteins associated with FAK after FAK inhibition treatment.

Protein	VS-5063			PF-573228			Combination		
	0.1 nM	1.0 nM	10 nM	0.1 nM	1.0 nM	10 nM	0.1 nM	1.0 nM	10 nM
ZYX	9.01	1.23	NF	NF	0.17	0.16	NF	NF	NF
ELAV1	0.57	0.61	—	0.72	0.51	0.28	1.2	0.63	NF
CAPN2	0.71	1.1	NF	0.68	0.65	0.79	0.53	1.11	NF
MAP2K2	—	—	—	—	—	—	0.74	—	NF
PPP2	0.54	0.57	0.22	0.31	0.37	0.34	0.31	0.38	NF
ARFGEF	NF	NF	NF	—	—	NF	NF	—	NF
SYNE1	2.65	2.65	NF	NF	2.87	3.7	3.76	2.6	0.43
DSP	100	—	—	—	—	—	—	—	—

Fold change = Inhibitor treatment/control.

*NF: Not found in the inhibited cells but found in the control cells.

**—: Not found in both inhibited cells and the control.

**Figure 4:**
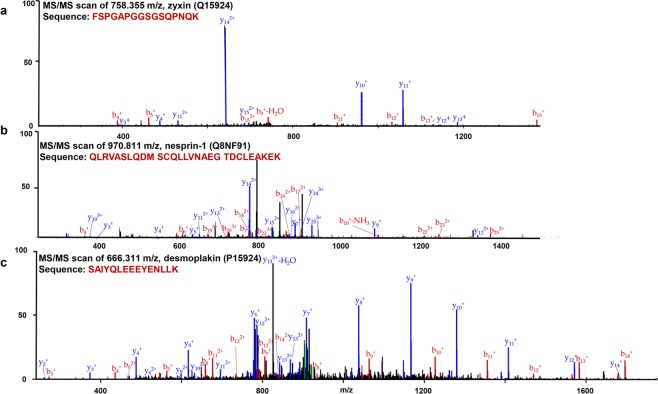
FAK’s binding partner identification. Data-dependent MS/MS sequencing scan of top peptides identifying: (**a**) zyxin, m/z 758.355. (**b**) nesprin-1, m/z 970.811. (**c**) desmoplakin, m/z 666.311.

Among the protein candidates, we selected three new, putative binding partners, zyxin, nesprin-1, and desmoplakin, based on how well their molecular functions and significance are known. Zyxin and desmoplakin were pulled down in both regular IP and EGF-stimulated IP in at least duplicate experiments. However, under the activation of FAK triggered by EGF, desmoplakin was more associated with active FAK than with the inactive form (4.72-fold increase in the abundance ratio). Unlike DSP, the interaction of zyxin and FAK was not directly linked to the activation of FAK under EGF stimulation (1.34-fold elevation in the abundance ratio). Nesprin-1 was not reproducibly immunoprecipitated in regular IP; nonetheless, under EGF stimulation, FAK interacted with nesprin-1 more substantially. Their spectrum MS/MS data are shown in Fig. 4 and Supplementary Table S1.

### Validation of selected FAK new binding partners by reciprocal immunoprecipitation and Western blot

**Figure 5:**
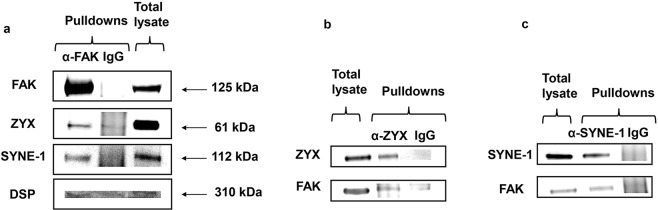
Reciprocal immunoprecipitation and Western blot analysis. (**a**) Immunoblot of proteins precipitated using anti-FAK antibodies. New associated proteins zyxin, nesprin-1, and desmoplakin were detected similarly to those present in total cell lysates but not in IgG immunoprecipitation controls. (**b**,**c**) Proteins immunoprecipitated from HCT-116 cell lysates using zyxin and nesprin-1 antibodies were immunoblotted on membranes using anti-FAK antibodies. FAK was detected in both pulldowns but not in IgG controls. Full-length blots are presented in Supplementary Fig. S5.

To validate the potent candidates, we performed Western blot analysis and reciprocal IP experiments. After stimulation with EGF, the total cell lysate HCT-116 was extracted, and anti-FAK IP was carried out. Western blots revealed protein bands corresponding to the new interactors zyxin, nesprin-1 and desmoplakin (Fig. 5). These bands were similar to those present in total cell lysates but not those in the IgG control lanes. For reciprocal IP, zyxin and nesprin-1 polyclonal antibodies were employed in HCT-116 cell lysate. Precipitated proteins were resolved by SDS-PAGE, and FAK bands were blotted by Western blotting in both IP experiments. Unfortunately, currently available antidesmoplakin antibodies were not valid for IP experiments; thus, we failed to conduct the reversed IP to confirm the interaction of FAK and desmoplakin.

**Figure 6:**
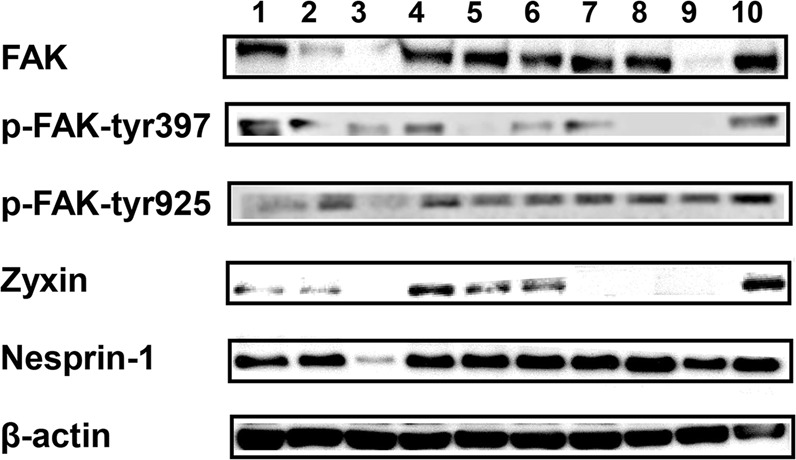
Western blot analysis from treatment with FAK’s inhibitors. 1. VS-6063 0.1 nM, 2. VS-6063 1 nM, 3. VS-6063 10 nM, 4. PF-573228 0.1 nM, 5. PF-573228 1 nM, 6. PF-573228 10 nM, 7. Combination 0.1 nM, 8. Combination 1 nM, 9. Combination 10 nM, 10. DMSO. Full-length blots are presented in Supplementary Fig. S8.

To clarify the relationship between FAK and protein candidates as well as to strengthen the evidence for previous experiment, we examined the protein expression level of zyxin and nesprin-1 in FAK inhibitor treatment cells by Western blot (Fig. 6). Zyxin and nesprin-1 levels were maximally reduced at the highest concentration of VS-6063. Upon treatment of two inhibitors, zyxin was completely attenuated which is in alignment with our previous proteomic data, suggesting a synergistic effect of two drugs. Intriguingly, nesprin-1 expression was paradoxically restored under concomitant treatment of two inhibitors. Noticeably, p-FAK-tyr925 correlated with the expression of nesprin-1, whereas p-FAK-tyr397 strongly associated with zyxin chaperone activity. This phenomenon may indicate a different inhibitory mechanism for the drugs.

### Zyxin knockdown impairs cancer-related pathways

To gain insight into the functional significance of candidates, we knocked out the genes encoding zyxin, nesprin-1, and desmoplakin by small interfering RNA (siRNA) transfection of HCT-116 cells (see supplementary materials). In all siRNA-based assays, we utilized well-characterized siRNA sequences directed against the candidate proteins and obtained knockdown of 85%, 80% and 75% at the protein level toward zyxin, nesprin-1 and desmoplakin, respectively (Supplementary Fig. S6)

One of the essential molecular hallmarks of the integrin activation state is FAK autophosphorylation. These early events that occur at tyrosyl residues FAK-397 and FAK-925 are significant indicators leading to several downstream signals. Interestingly, we observed striking reduction in p-FAK-tyr397 and p-FAK-tyr925 levels in all depleted-cells, with the most decreasing of p-FAK found at zyxin-transfected cells.

Autophosphorylation of FAK on tyrosine 397 is one of the early events that occur after the clustering of integrin that leads to many critical downstream signals. Compared to transfection with control siRNA, transfection with either zyxin or nesprin-1 siRNA resulted in a significant reduction in the phosphorylation of ERK, although the effect was weaker with nesprin-1 siRNAs. Nevertheless, desmoplakin underexpression did not remarkably influence p-ERK levels.

**Figure 7:**
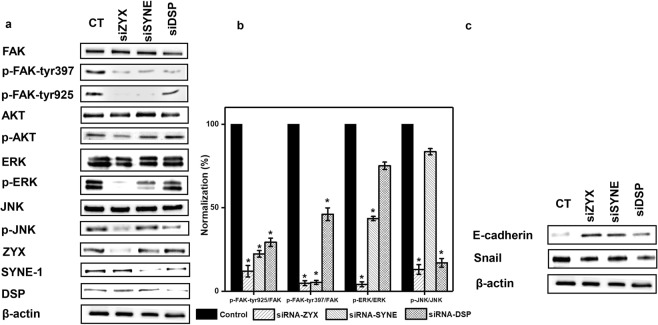
siRNA-mediated knockdown of zyxin, nesprin-1 and desmoplakin related to the ERK, AKT, JNK, EMT pathways. HCT-116 cells were transfected with the indicated siRNAs and lysed. Phosphorylation of FAK (tyr397 and tyr 925), ERK, AKT, and JNK were measured by Western blot with corresponding phosphor-site-specific antibodies. For quantification, the phosphorylation signal of each protein was first normalized to the total protein signal. The highest phosphorylation value (control) was used as a reference and set to 100% to which all other values were correlated. The bars show the mean ± SD. The significance of each value against the corresponding control is shown (n ≥ 3, **p < 0.01). Full-length blots are presented in Supplementary Fig. S9.

Next, we addressed the phosphorylation state of the JNK cascade. The p-JNK protein was lower in all zyxin- and desmoplakin-silenced cells than in control cells. Moreover, nesprin-1 transfected cells demonstrated only a tenuous decrease in JNK phosphorylation. We also examined the correlation of gene knockdowns against the AKT pathway. However, a modest reduction was exclusively observed in zyxin-transfected cells, whereas the protein levels remained unchanged in nesprin-1 and desmoplakin siRNA-mediated cells compared with those in control cells (Fig. 7a,b).

### Knockdown of zyxin and nesprin-1 inhibits migration and invasion

EMT is a process by which epithelial cells lose their cell polarity and cell-cell adhesion and gain migratory and invasive properties to become mesenchymal cells. The EMT process in HCT-116 cancer cells is characterized by changes in morphology, loss of epithelial protein marker E-cadherin and gain of the mesenchymal protein marker snail. Our siRNA-mediated silencing of zyxin and nesprin-1 resulted in a moderate increase in the E-cadherin protein level accompanied by a reduced level of snail protein, indicating that knockdown of these proteins restrained cell migration and invasion (Fig. 7c).

**Figure 8:**
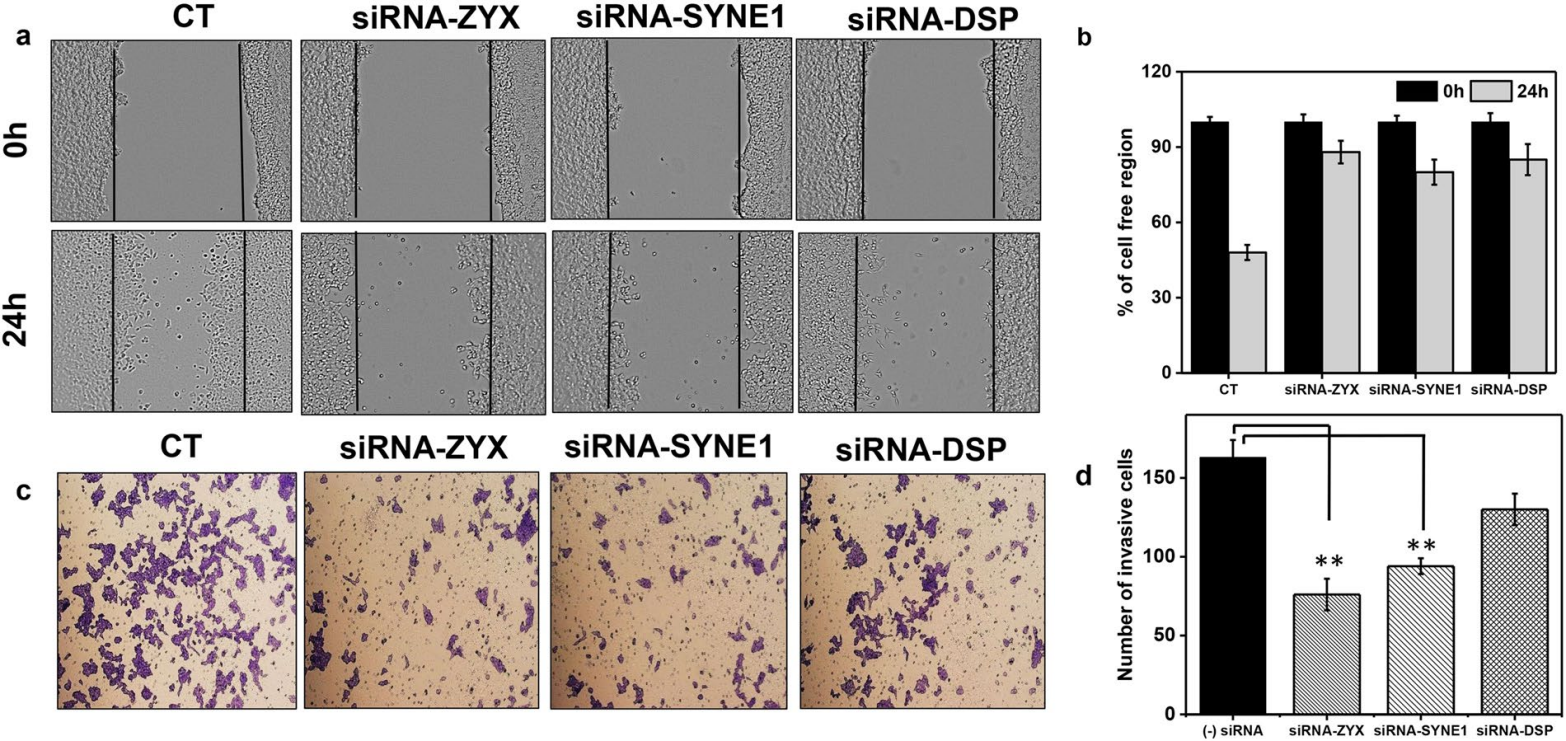
Gene knockdown impaired cell migration and invasion. (**a,b**) Wound-healing assay. HCT-116 cells transfected with the indicated siRNAs were allowed to reach confluence. A scratch was produced at 0 h (upper panel), and the closed gap was measured after 24 h (lower panel). The chart shows the percentage of the cell-free region. The bar shows the mean ± SD (n ≥ 3, **p < 0.01). (**c**,**d**) Matrigel invasion assay. HCT-116 cells were transfected with the indicated siRNAs, detached, and seeded on transwell chambers coated with Matrigel. Cells that invaded through the membrane were counted under an inverted microscope. The bar shows the mean ± SD. The significance of each value against the corresponding control is shown (n ≥ 3, **p < 0.01).

Compared with control cells, siRNA-treated cells appeared to be altered morphologically with changes in cell shape together with a decrease in internalization from cell-cell contact (Supplementary Fig. S7). The morphological transformation of siRNA-treated cells consequently impacted their migratory and invasive capacity. To better understand this transformation, we performed a wound-healing assay (see supplementary materials) in which a monolayer of cells is damaged by producing a scratch of standard width and the closure of this wound by cells migrating toward each other from both sides. After 24 h, control siRNA-transfected cells had filled the gap by 50%, whereas with zyxin, nesprin-1 and desmoplakin knockout cells, an open space was still observed between the wound edges, which was highly marked in zyxin-depleted cells. In parallel with the migration assay, we conducted a Matrigel invasion assay to elucidate the impact of zyxin, nesprin-1, and desmoplakin on the invasive behaviors of HCT-116 cancer cells. In brief, a Transwell chamber was used to determine the movements of cells through a Matrigel-coated membrane, mimicking the early step of tumor invasion (see supplementary materials). As shown in Fig. 8a,b, after a 24-h incubation, the number of cells that invaded through the artificial basement membrane was 163 ± 11 cells/field in the control group, 76 ± 10 cells/field in the zyxin-depleted group, 94 ± 5 cells/field in the nesprin-1 siRNA-mediated group and 130 ± 9 cells/field in the desmoplakin knockdown group (Fig. 8c,d). These results indicate a considerably lower number of invasive cells through the Transwell membrane in the zyxin depletion group, followed by the nesprin-1 transfected group compared with those in the control and desmoplakin depletion groups.

Taken together with the migration assay findings, these results provide evidence that the above-mentioned proteins are involved in the regulation of cell-matrix adhesion, cell migration, and invasion.

### ELISA results revealed zyxin and nesprin-1 as potential diagnostic targets for cancers

**Figure 9:**
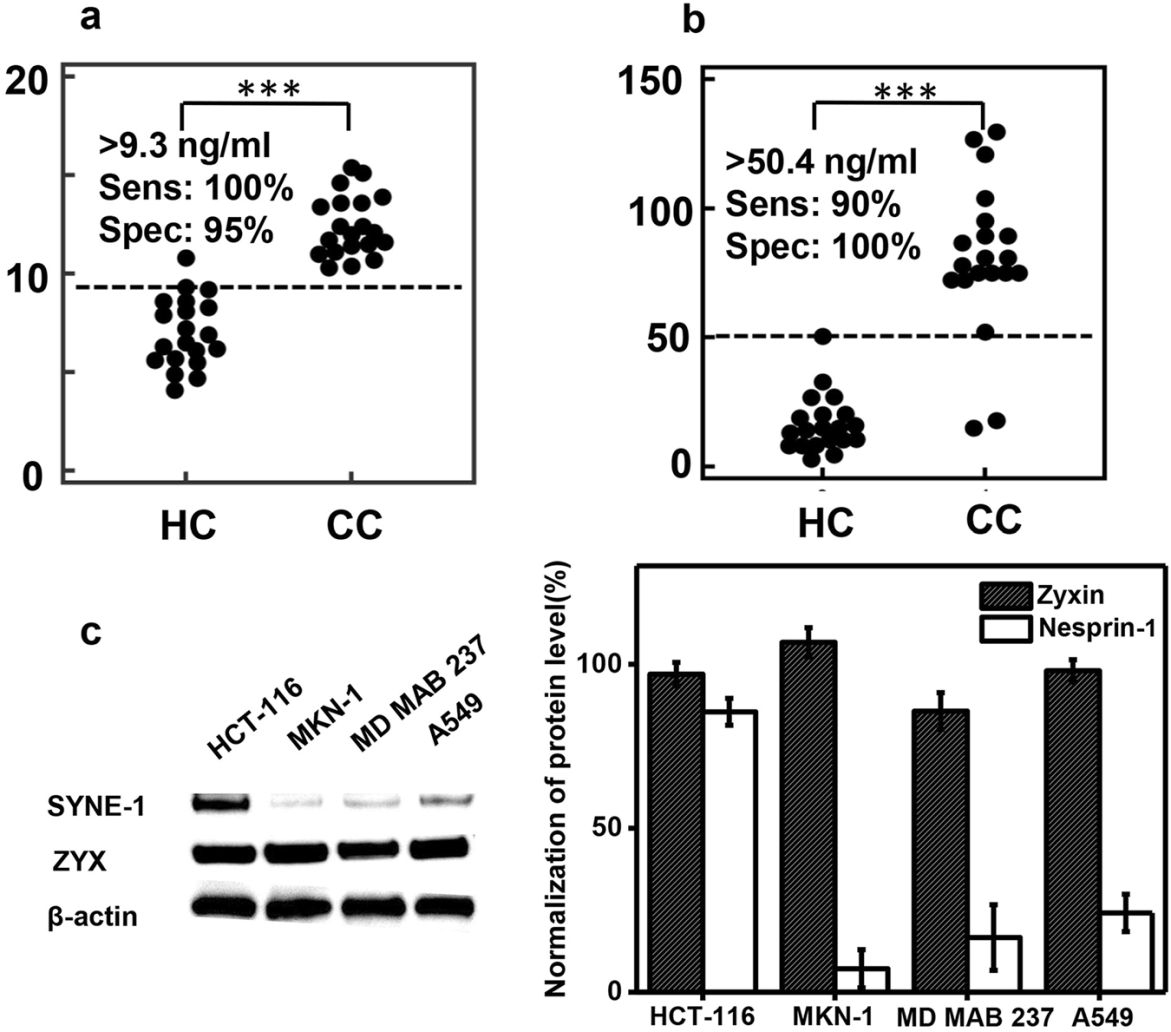
Zyxin and nesprin-1 as biomarkers for colon cancer. Cutoff values of biomarker concentrations (ng/ml) in samples from patients with colon cancer using ELISA assays. (**a**) Detection against zyxin. (**b**) Detection against nesprin-1. Significant difference vs. control cohort ***p < 0.001. (**c**) Western blot analysis of zyxin and nesprin-1 protein level in various cancer cells. HCT-116: colon cancer; MKN-1: gastric cancer; MD MAB237: breast cancer; A549: lung cancer. Full-length blots are presented in Supplementary Fig. S10.

The ultimate purpose of our study is to target protein candidates in terms of cancer diagnostics or therapeutics. Early intervention in cancer dramatically increases the chances for successful treatment. Pathological diagnosis is particularly crucial for colon cancer, as at the late stage, it spreads outside the colon or rectum, leading to extremely low survival rates. In this work, ELISA assays were performed using 20 sera samples from patients with colon cancer and 20 healthy donor samples to quantify the protein levels of zyxin and nesprin-1. Then, we generated ROC curve analysis for zyxin and nesprin-1 detection. The AUC values obtained from zyxin and nesprin-1 were 1 and 0.97, respectively, and no significant difference between these values suggested that both proteins could be excellent diagnostic factors. From the ROC curves, the optimal cutoff concentration values of 9.3 and 50.4 ng/ml were determined for zyxin and nesprin-1, respectively, to exclude any background signals produced by control groups (Fig. 9a,b).

Additionally, we performed Western blot analysis and observed that nesprin-1 expression was significantly high in colon cancer compared to those of gastric cancer, breast cancer, and lung cancer (Fig. 9c). Although the protein level of zyxin was not distinguishable in colon cancer among others, taken together with ELISA results, these two proteins reveal promising potency to contribute to cancer diagnosis and therapeutic development.

## Discussion

Our study presents a functional proteomic approach to determine the identity of proteins that may directly or indirectly interact with FAK. To classify potential binding partners of FAK, we set up a proteomic screening using immobilized FAK fused to agarose bead-anti-FAK antibody as bait and HCT-116 cell extract as a source of potential substrate. The N-terminal in the structure of FAK participates in multiple protein-protein interactions via mediation by growth factors.

We compared the IP obtained from untreated HCT-116 cells and EGF (epidermal growth factor) stimulated HCT-116 cells because it is evidenced that the proliferation and growth-promotion of experimental colon cancer models are efficiently acquired by the expression of EGF^44^. Therefore, in complementary IP, we triggered cells with EGF to recruit a number of interesting binding proteins or enhance their intensity in multimeric protein complexes. Our IP SDS-PAGE silver stain revealed the enhancing intensity of the upper and lower bands to FAK in the EGF stimulation panel. However, a band-to-band comparison analysis strategy was not implemented since our purpose focused on non-target protein profiling. Thus, the entire cell lane except for the IgG heavy chain band was excised and processed for further experiments. In addition, our gel cutting method allowed us to include proteins that may not be visualized by staining. By IP-MS analysis and applying stringent filtering criteria, we were able to map a total of 101 proteins with very high confidence. Importantly, FAK was the most prominent protein identified by our method in terms of the SEQUEST score, X_corr_ value, and number of matching peptides. These proteins were classified according to their relevant processes and molecular function. Notably, several interactors primarily ligate to proteins or RNA and are involved in metabolic processes or regulation of the biological system.

In the stimulated cell dataset, ingenuity pathway analysis predicted that EGFR is one of the most activated upstream regulators by Z-core. A mechanistic network generated for EGFR in this experiment demonstrated that it might exert its effects on the observed proteins by interacting with EGF (as expected) and through coactivators and other regulatory molecules in networks in which FAK (PTK2) is a central node (Supplementary Fig. S11). Among the networks, other central nodes, such as TP53 and CCND1, are previously discovered therapeutic targets^45–47^. Many known FAK binding partners, including FAK itself, were strongly enriched in the protein matrix after EGF stimulation. Intriguingly, 18 proteins were steadily pulled down in stimulated cells, whereas their presence in regular IP was either stochastic or nonexistent. Of those 18 proteins, a vast majority are cytoskeleton members, such as actinin, filamin, and myosin, revealing that the activation of FAK under EGF stimuli regulates the stability and dynamic changes in the cytoskeleton.

To better comprehend the interacting network of FAK, as the activation status of FAK may affect protein-protein interactions, we inactivated FAK by treating HCT-116 cells with FAK inhibitors. In-solution digestion coupled with LC-MS/MS was utilized to analyze the cell proteome. We noticed several proteins associated with FAK in a drug dose-dependent manner. For instance, ELAV1 coexists with FAK in the recruitment of GRB2 protein when p-FAK-tyr397 occurs^48,49^. Binding of human CAPN2 protein and FAK increases migration of Nb7-cells bound to fibronectin in culture^50^. MAP2K2 associates with MAPK1 in a multimolecular complex linked with FAK and Paxillin^51,52^. Additionally, inhibition of active FAK reduces activation of AKT, causing the decreased binding of AKT and PPP2C protein^53^. ARHGEF binds to CD44 protein, resulting in cytokine production and breast tumor progression, whereas CD44 binds to FAK in the context of cellular activation in Hela-CD4 cells^54,55^.

Interestingly, these proteins reacted differently to VS-6063 and PF-573228 via the fold change between drug-treated cells and control cells. VS-6063 and PF-573228 are both small molecules targeting the ATP-binding site at tyrosine 397 demonstrated by the reducing p-FAK-tyr397 level in both drug treatments. While VS-6063 seemingly affected on both tyrosines 397 and 925, PF-573228 selectively targeted at tyrosine 397. In our study, VS-6063 performed a more inhibitory potency than that of PF-573228. This may be explained by the N-methyl-benzamide group on VS-6063 being more reactive and hydrophilic compared to the quinoline group on PF-573228, making VS-6063 more accessible to the ATP-binding pocket. ATP-binding sites share consensus sequences and structural domains across many different tyrosine residues on FAK, resulting in multiple phosphorylation sites on the kinase domain. It is therefore plausible to postulate the potency of VS-6063 in our work. However, these two clinical trial drugs may confound the data interpretation in colon cancer cells due to their current selectivity to other cancers^56,57^. The careful evaluation of pharmacokinetics and pharmacodynamics will be required to fully understand the role of FAK inhibitors in colon cancer.

To determine the relevant biological significance of these proteins in the mediation of numerous intracellular signaling and regulatory pathways, we knocked out genes to clarify their functions in HCT-116 cells. Identifying the signaling pathways through which these proteins are involved in these activities is imperative. The ERK pathway has diverse effects on apoptosis, growth and cell survival^58^ and is activated by several mechanisms. The JNK pathway is associated with apoptosis, neurodegeneration, cell differentiation and cytokine production mediated by AP-1 (activation protein 1), such as RANTES, IL-8 and GM-CSF^59^.

In this study, siRNA-mediated zyxin and nesprin-1 knockdown resulted in decreased p-FAK-tyr397 followed by p-ERK levels, whereas silencing of desmoplakin led to a reduction in p-JNK. Although the autophosphorylation of FAK was impaired by zyxin, SYNE-1 siRNA and DSP-siRNA did not produce noticeable changes in FAK phosphorylation. Similarly, we did not detect any remarkable change in the AKT signaling pathway in any siRNAs. However, FAK inhibition was previously correlated with zyxin in our in-solution digestion, and the depletion of zyxin induced the inactivation of FAK via reduced p-FAK levels, strengthening a direct association. Nonetheless, the precise mechanism by which proteins govern one another needs further in-depth studies. Such similar schemes should be applied for nesprin-1 and desmoplakin.

We revealed that the depletion of zyxin and nesprin-1 significantly controlled the regulation of cell-cell contact integrity and motility. As demonstrated in several studies, zyxin colocalizes with VASP protein at cell-cell junctions and cooperates with actin filament formation and binding. This connection with actin filaments establishes robust intercellular adhesion. Integrins are the primary protein at focal adhesion plaques and connects the ECM to actin filaments that regulate cell adhesion, spreading and motility. Therefore, zyxin may likely act as a signaling molecule that transmits information from adhesion plaques to the cytoskeleton system via FAKs.

Nesprins link the nucleus to the cytoskeleton and are crucial components that transduce mechanical signals from the cytoskeleton to the nuclear lamina^60^. King *et al*. proved that depletion of nesprin-1 reduced the migration of endothelial cells into a cell-free area^61^. In a study by Chancellor *et al*., nesprin-1 regulated migration through focal adhesion, and its depletion was associated with a number of integrin-based focal adhesion molecules that remodel the structure of cell adhesion^62^. Herein, the decrease in cell spread upon nesprin-1 knockout was consistent with previous studies.

Belong to one of core member of the desmosome, desmoplakin binds with two other desmosomal component in the complex cadherin-armadillo complex, thus form the intermediate filament network to the junctional plaque^63,64^. Recent studies show evidence that FAK is a possible regulator of blood testis barrier integrity^65^. Indeed, it was evidenced that phosphorylation of FAK at the residue tyrosine 397 structurally interacted with β1-integrin, c-Src, vinculin in sertoli-germ cell cocultures to establish anchoring junctions^66^. Such activities, including basal ectoplasmic specialization together with tight junctions, and gap junctions utilize F-actin filament where FAK is a regulator of this cytoskeletal organization, suggesting a possible interaction between FAK and the desmosome^67^. The study in blood testis barrier proposed a molecular regulatory pathway to maintain the immunological barrier in which desmosome, gap junctions are participating in concert with FAK-Src signaling cascade^68^.

According to Yang *et al*., desmoplakin functions as a tumor suppressor by inhibition of the Wnt/β-catenin signaling pathway in human lung cancer^69^. In our study, desmoplakin knockout significantly reduced p-JNK, suggesting a different mechanism for various cancer cell lines, although further investigation is needed.

Although desmoplakin depletion did not remarkably affect migration and invasion in our results, the additional evidence supporting the function of DSP in colon cancer cells was interesting as desmoplakin provided insights into promising cancer therapeutic targets.

Our final purpose is to target protein candidates for the diagnosis, prognosis or treatment of colon cancer. Hence, we performed ELISA assays on patient samples to evaluate differences in the concentration of zyxin and nesprin-1 relative to that in healthy controls. In biomarker discovery, sensitivity and specificity are two gold standard criteria to evaluate the validation of candidates. Accordingly, the sensitivity and specificity for diagnosing colon cancer from healthy controls using zyxin were 100% and 95%, respectively. The values obtained for nesprin-1 were 90% and 100%, respectively. Both zyxin and nesprin-1 were more highly expressed in patient samples than in control samples. Indeed, Kim *et al*. verified zyxin as a marker candidate for non-small-cell lung cancer in their study^70^, however, the Western blot analysis of zyxin protein level found in our colon cancer cells HCT-116 was comparable to that of A549- a non-small-cell lung cancer model suggesting zyxin might act as a generic cancer therapeutic target. In contrast, with the significant elevation of nesprin-1 in HCT-116 cells among other cancers, this protein could be nominated as a promising target for colon cancer.

In summary, we identified FAK-interacting proteins from diverse classes of proteins in colon cancer cell line HCT-116. Among the binding partners, selected candidates were validated, and their relevant biological functions were considered. The findings of the present study might shed light on pathological aggregation occurring in cancers, especially for colon cancer cells. The features of zyxin and nesprin-1 identified herein provide a new perspective for future studies on these proteins that may act as therapeutic or diagnostic targets.

## Materials and Methods

### Cell lysis and immunoprecipitation

HCT-116 cells were detached using trypsin-EDTA (TE) and washed twice with cold PBS. Cells were lysed using IP lysis buffer (Pierce, ThermoFisher Scientific, Waltham, MA, USA) and centrifuged at 12,000 g for 15 min at 4 °C. The supernatant was collected and stored at −20 °C. A bicinchoninic acid kit (ThermoFisher Scientific) was used to measure protein concentrations. For IP, lysate containing 1500 µg total protein was mixed with either 10 µl anti-FAK antibody (Cell Signaling Technology, MA, USA) or normal rabbit IgG (Merck, NJ, USA) as a negative control in a total volume of 500 µl. Before IP, the lysates were precleared with 10 µl agarose slurry control to remove nonspecific binding between proteins and beads for 3 h at 4 °C with end-over-end rotation. Pierce™ Protein A/G Agarose beads (ThermoFisher Scientific) were preblocked with 1% BSA in PBS under the same conditions as those used for lysate preclearance. Then, antibodies were added to the clarified lysate for 2 h at RT with end-over-end rotation, followed by incubation with preblocked agarose slurry in PBS overnight at 4 °C. Beads were washed 5 times with 500 µl PBS-Tween 0.1% and 3 times with 500 µl PBS, resuspended in 40 µl 2X Laemmli Sample Buffer (Merck, NJ, USA) and boiled for 10 min at 95 °C. Next, proteins were separated on a 10% SDS-PAGE gel and visualized with an EzStain Silver kit (ATTO, Japan).

### Western blot analysis and reciprocal immunoprecipitation

Protein extracts (25 µg) prepared with Pierce™ IP Lysis buffer were resolved by 10% SDS-PAGE and transferred onto a nitrocellulose membrane (Pall Corporation, FL, USA). Membranes were blocked with PBS containing 0.02% Tween 20 and 5% nonfat dry milk and blotted overnight with primary antibodies against proteins of interest (anti-FAK, anti-p-FAK-tyr-925, anti-p-FAK-tyr397, anti-AKT, anti-p-AKT, anti-ERK, anti-p-ERK, anti-JNK, anti-p-JNK (Cell Signaling Technology, Danvers, MA, USA) and anti-β-actin from Abfrontier (San Diego, USA). HRP-conjugated goat anti-rabbit IgG purchased from Santa Cruz Biotechnology Inc. (Santa Cruz, CA, USA) was used as the secondary antisera at a 1:5000 dilution. Protein bands were visualized using a chemiluminescence imaging system, Ez-Capture MG (ATTO, NY, USA).

Reciprocal IP was performed using HCT-116 lysates (1500 µg total protein) and antibodies against zyxin, nesprin-1, and desmoplakin (Abcam, Cambridge, UK). Immunoprecipitated proteins were resolved by SDS-PAGE, and FAK was detected by Western blotting.

### Epithelial growth factor stimulation and FAK inhibitor treatment

Once HCT-116 cells were confluent at 2 × 10^7^ cells, these cells were starved overnight in serum-free RPMI 16 and stimulated with or without 10 ng/ml hEGF (Merck, NJ, USA) for 30 min at 37 °C. After two rinses with cold PBS, cells were lysed, and protein concentrations were measured.

A total of 2 × 10^7^ confluent HCT-116 cells at the starvation stage were incubated with FAK inhibitors VS-6063 and PF-573228 (selleckchem.com, Houston, TX, USA) with a working range of 0.1-10 nM for 24 h, harvested, rinsed twice with cold PBS, and lysed. Live cells were counted by a Countess II FL automated cell counter (Life Technologies, Carlsbad, CA, USA).

### In-gel digestion and in-solution digestion

Gel lanes were diced into small pieces (1 × 1 mm) and subjected to in-gel reduction and alkylation with 10 mM DTT and 55 mM iodoacetamide, respectively. Gel pieces were washed with 100% ACN, dried and rehydrated with 15 ng/ml trypsin (Pierce, ThermoFisher Scientific) in 10 mM ammonium bicarbonate containing 10% (vol/vol) acetonitrile for 30 min, and the digestion continued overnight at 37 °C. Tryptic peptides were extracted with 1:2 (vol/vol) 5% formic acid/acetonitrile buffer, concentrated with vacuum centrifugation and desalted using a C18 Spin Column according to the manufacturer’s protocol (Pierce, ThermoFisher Scientific).

For in-solution digestion, 100 µg total cell lysates from the abovementioned FAK inhibitor treatment was freeze-dried for 3 h, reconstituted in 100 µl 6 M urea, and reduced and alkylated in 200 mM DTT and 100 mM IAA, respectively. The digestion continued with the addition of 2 µg trypsin and incubated overnight at 37 °C. The samples were cleansed using a C-18 Spin column.

### Nano-LC-MS/MS analysis

Samples were analyzed on an EASY-nLC1000 system (ThermoFisher Scientific) coupled to an LTQ-Orbitrap Velos pro (ThermoFisher Scientific, Sunnyvale, CA, USA). Peptides for analysis were loaded via an Acclaim PepMap 100 trap column (100 µm × 2 cm, nanoViper, C18, 5 µm, 100 Å, Thermo Scientific), and subsequent peptide separation was performed on an Acclaim PepMap EASY-Spray analytical column (75 µm × 15 cm, nanoViper, C18, 2 µm, 100 Å, Thermo Scientific). For each liquid chromatography tandem mass spectrometry (LC-MS/MS) analysis, peptides were loaded on a precolumn with microliter pickup. Peptides were separated at a flow rate of 250 nL/min using mobile phase acetonitrile with the addition of 0.1% formic acid in a gradient of 5–40% over 120 min. Eluted peptides were examined with an Orbitrap mass spectrometer using a spray voltage of +1.8 kV. A full scan from m/z 300 to 2000 at a resolution of 60,000 was acquired. Top N mode followed by a ten data-dependent acquisition of MS/MS scan method was applied using CID with normalized energy of 35 eV. Dynamic exclusion for previously fragmented precursor ions was used with the following parameters: exclusion time 180 s, repeat count 1, repeat duration 30 s, exclusion mass width 10 ppm, exclusion size 500 m, signal threshold 5000, and isolation width 1 m/z. Singly charged species were excluded from fragmentation.

### Protein identification

The acquired MS/MS data were searched using the search engine Proteome Discoverer v2.2 (ThermoFisher Scientific) against the SEQUEST algorithm with amino acid sequences in the SwissProt database (2017). All search results were run through Percolator for scoring. Search parameters were set as follows: enzyme, trypsin; precursor ion mass tolerance, 10 ppm; fragment ion mass tolerance, 0.6 Da; maximum missed cleavages allowed 2; and oxidation (+15.995 Da) and carbamidomethyl (+57.021 Da) for variable modification. The peptide matching criteria was a SEQUEST HT score >1. The peptide matching criteria was a cross-correlation score (X_corr_) >1.2 for +1 peptides, >2.2 for + 2 peptides, and >3.0 for +3 peptides. Other considered criteria included the number of unique peptides per protein ≥2 and molecular signaling interactions. All proteins passed a threshold of less than a 5% false discovery rate.

### ELISA assays

Twenty serum samples were collected from patients with colon cancer (n = 20, 11 males and 9 females) at the Department of Laboratory Medicine, Severance Hospital and Yonsei University College of Medicine (Seoul, Korea). The mean age was 62 years old (range 40–76). The mean carcinoembryonic antigen (CEA) value was 2.317. Serum collection was performed and the experiment was approved by the institutional review board (IRB) of Severance Hospital (IRB no. 4-2017-0321). Sera from 20 healthy controls (HCs, n = 20, 10 males and 10 females) were obtained from the Korea University Medical Center (Seoul, Korea). The HC sample collection procedure was in compliance with the Declaration of Helsinki, and the study was approved by the institutional review board (IRB) of the College of Medicine and Korea University (IRB no. KUGH12118-005). All participants gave written informed consent before participation in the study.

A Human Nesprin 1 ELISA kit and Zyxin ELISA from Mybiosource.com (San Diego, USA) were used according to the manufacturer’s protocols. Optical density values were recorded at 490 nm using a microplate reader (Bio-Rad, CA, USA).

### Statistical studies and ingenuity pathway analysis

At least three independent experiments were performed for each assay. Data are presented as the mean ± SD. Densitometric quantification was performed using a CS Analyzer (ATTO, Japan). Differences between groups were determined by ANOVA test (Origin 2017, USA). P-values represent the statistical significance of the differences between treatment and control conditions. The significance of each value is shown with respect to the corresponding control values. The p-values are defined as follows: *p < 0.05, **p < 0.01, and ***p < 0.001. Sensitivity and specificity analyses were performed, and receiver operating characteristic (ROC) curves and areas under the curve (AUCs) were calculated with MedCalc v.15.2.1 software (MedCalc, Ostend, Belgium).

Bioinformatic study was performed to analyze differentially expressed genes under EGF stimulation followed by FAK-IP. Datasets containing gene identifiers and corresponding expression values (fold change) derived from Proteome Discoverer were uploaded into Ingenuity Pathway Analysis software. Core analysis interpreted the data in the perspective of biological processes, diseases, pathways, and networks. Genes differentially expressed with p < 0.05 were overlaid onto global molecular networks in in the Ingenuity pathway knowledge base (IPKB). The algorithm generated the most relevant networks based on their connectivity and most prevalent functional groups were named on networks accordingly. Canonical pathways identified from core analysis presented the specific genes within the networks under EGF stimulation.

## Supplementary information

Supplementary information

Supplentary info of IgG control protein

## Data Availability

All experiments performed and data analyzed during this study are included in this article and its Supplementary Information Files.
