# Supplementary material for: Identification of new binding proteins of focal adhesion kinase using immunoprecipitation and mass spectrometry
Source: Sci Rep. 2019 Sep 9;9:12908. doi: 10.1038/s41598-019-49145-6 (PMC6733923; doi:10.1038/s41598-019-49145-6)
Supplement: Supplementary file 1 — Supplementary information [file 41598_2019_49145_MOESM1_ESM.pdf]

Supplementary information

Identification of new binding proteins of focal adhesion kinase using immunoprecipitation and mass spectrometry

Binh Thanh Nguyen, Jae-Chul Pyun, Sang-Guk Lee, Min-Jung Kang

Methods

Cell lines, and cell cultures

Human colon cancer cell line HCT-116 was purchased from Korea Cell line bank (KCLB, South Korea) and routinely cultured in RPMI-1640 media supplemented with 10% (v/v) heated-inactivated fetal bovine serum and 1% (v/v) penicillin/streptomycin onto plastic culture dishes of 100 mm (Corning Life Science, Acton, MA, USA) at a density of $2×10^6$ cells/cm^2. The cultures plates were maintained in a humidified atmosphere of 5% CO_2 and 37°C. Prior to the treatments, the medium was replaced with serum-free medium for 24 h to synchronize the cells at the starving stage.

siRNA transfection

HCT-116 cells were cultured in six-well plates. Confluent cells were transfected with Zyxin, Nesprin-1, desmoplakin siRNA or control siRNA using Lipofectamine RNAiMAX (Invitrogen, Carlsbad, CA, USA) according to the manufacturer instructions. Three siRNAs including [Zyxin (sense: CUCCUAAGUUUACUCCUGU (dTdT), antisense: ACAGGAGUAAACUUAGGAG (dTdT)]; [Nesprin (sense: CACGAAGGCAACCAGAGA (dTdT), antisense: AUCUCUGGUUGCCUUCGUG (dTdT)]; [Desmoplakin (sense: CUGUUCAGGAGGCCUACAA (dTdT), antisense: UUGUAGGCCUCCUGAACAG (dTdT)]

After 6h incubation at 37°C, transfection media was removed and RPMI-1640 media supplemented with 10% FBS was added. After 48 h, cells were washed in cold PBS, harvested and lysed for further experiments.

Wound-healing assay

Fully confluent cells in six-well plates were starved in serum-free media for 24 h. A sterile 100- µl pipette tip was used to initiate a wound by creating a scratch on the cell monolayer. Cell debris was removed by extensive washing with PBS. The culture media was replaced with growth

media (5% FBS), containing zyxin, nesprin-1, desmoplakin siRNAs and cells were allowed to migrate into the wound area for 24 h at 37 ºC followed by image captures at a magnification of 100x.

Matrigel-invasion assay

Cell invasion was evaluated using transwell chambers with an 8 µm pore size. Cells (1×10^5 cells/well) were suspended in 100 µl of RPMI-1640 serum-free media and added to the upper chamber that had been coated with Matrigel (BD Bioscience, USA), followed by the addition of serum-free RPMI-1640 medium containing Zyxin, Nesprin-1, Desmoplakin siRNAs. The lower cavity of the transwell was filled with 600 µl of 10% FBS medium containing fibronectin (5 µg/ml) as a chemoattractant. After incubation in a humidified incubator for 24 h, cells in the upper chamber were removed with a cotton swab. Cells in the bottom chamber were fixed with 4% formaldehyde and permeabilized with 100% methanol, followed by staining with Giemsa (Merck, USA) for 15 min at RT. Cells were counted using a fluorescence microscope (Nikon Eclipse TE 2000-U) by randomly selecting five fields per membrane. The invasiveness of cells was expressed as the mean number of cells that invaded the lower capacity of the chambers. Each experiment was performed in triplicate.

Fig. S1a IP-1D Gel-LC/MSMS analysis of FAK interaction in human colon cancer cell HCT-116. a) Silver staining of immunoprecipitated proteins. Control IP referred non-stimulated cells. b) Venn diagram indicates the number of identified proteins from control IP and EGF-stimulated IP.

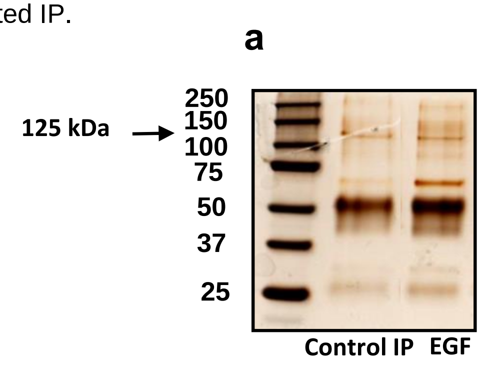

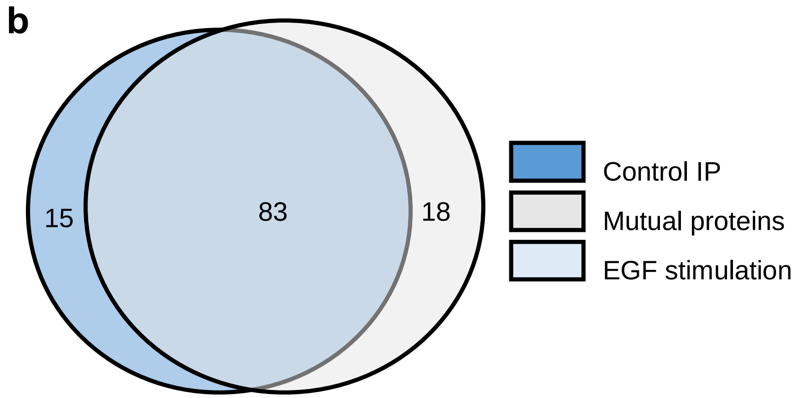

Fig. S1b. Full blots of Figure 1a in the main text

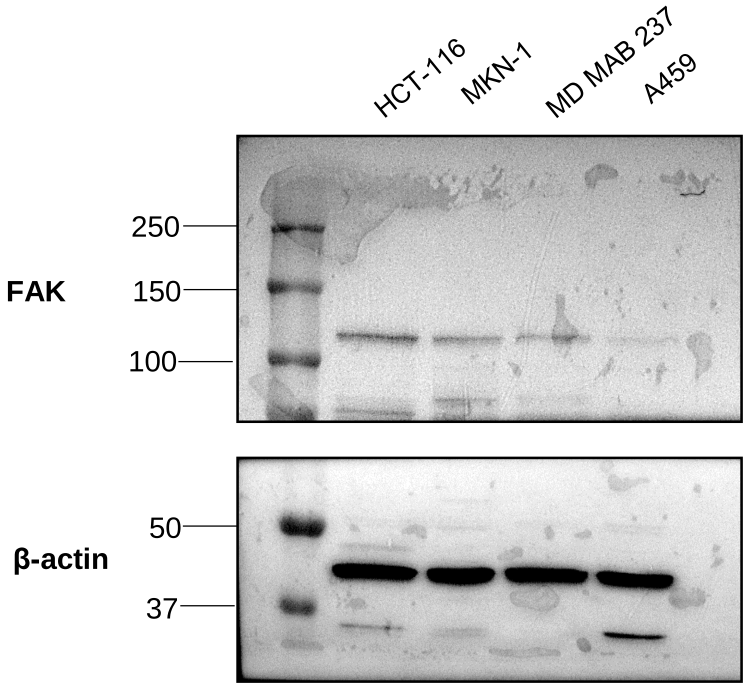

Fig. S2 FAK identification by tandem mass spectrometry. MS/MS sequencing of peptides identifying FAK. The MS full scan is shown followed by data-dependent MS/MS sequencing scan of top peptide candidate

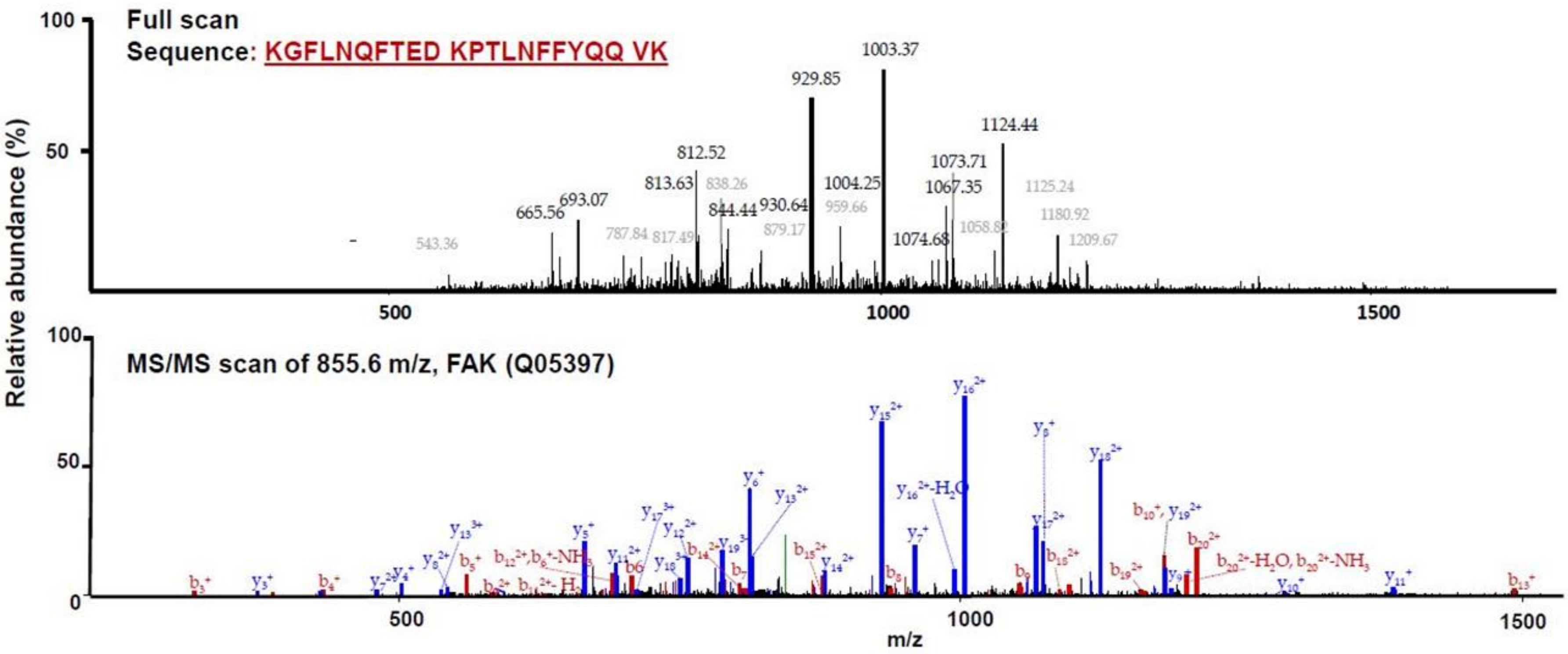

Fig. S3 Protein identification using in-solution digestion of whole cell lysate and in-gel digestion after immunoprecipitation.

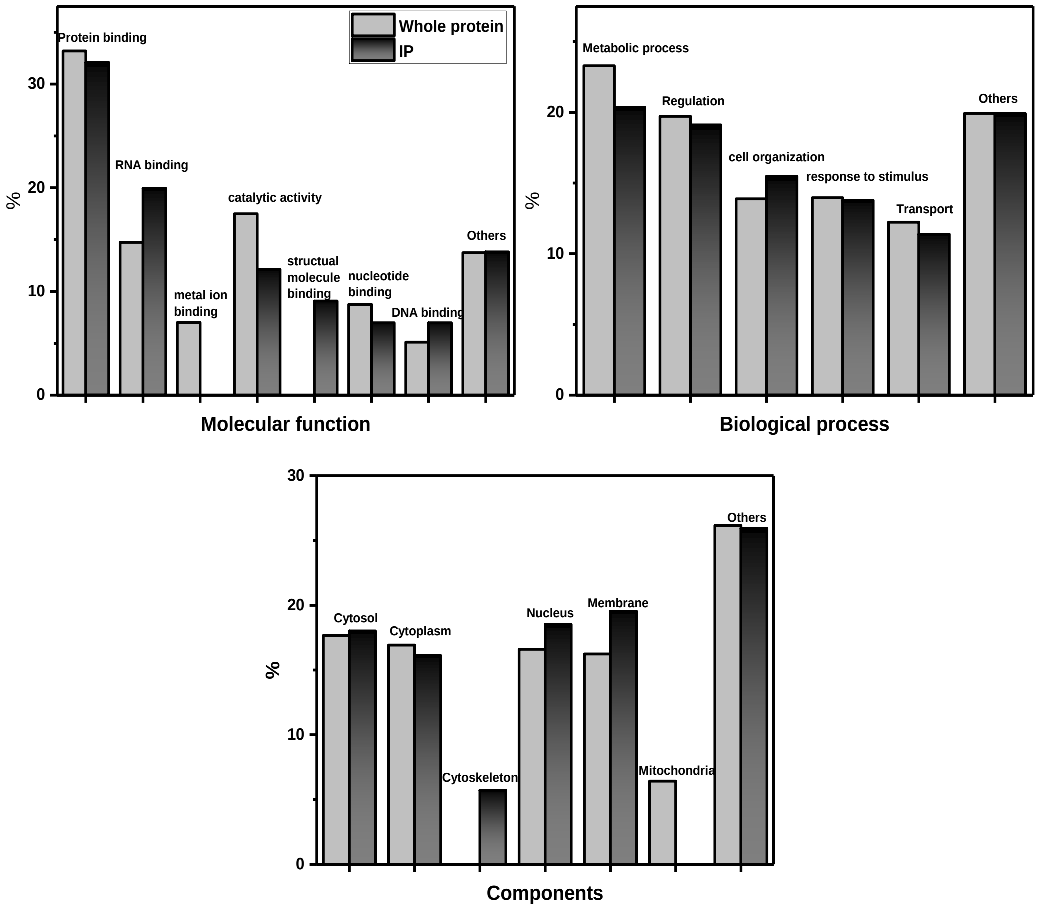

Fig. S4 Treatment of FAK inhibitors in HCT-116 cells. Identified proteins using in-solution digestion coupled with LC-MS/MS analysis

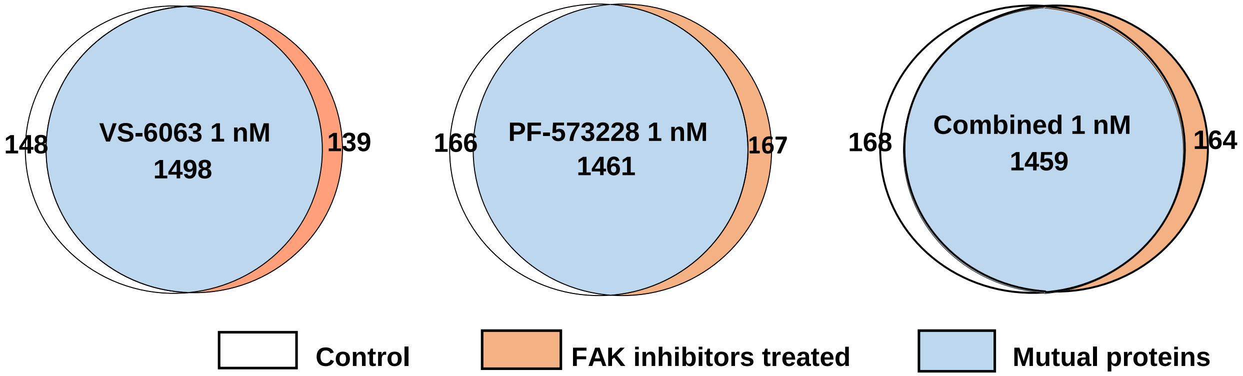

Fig. S5. Full blots of reciprocal IP from figure 5 in the main text

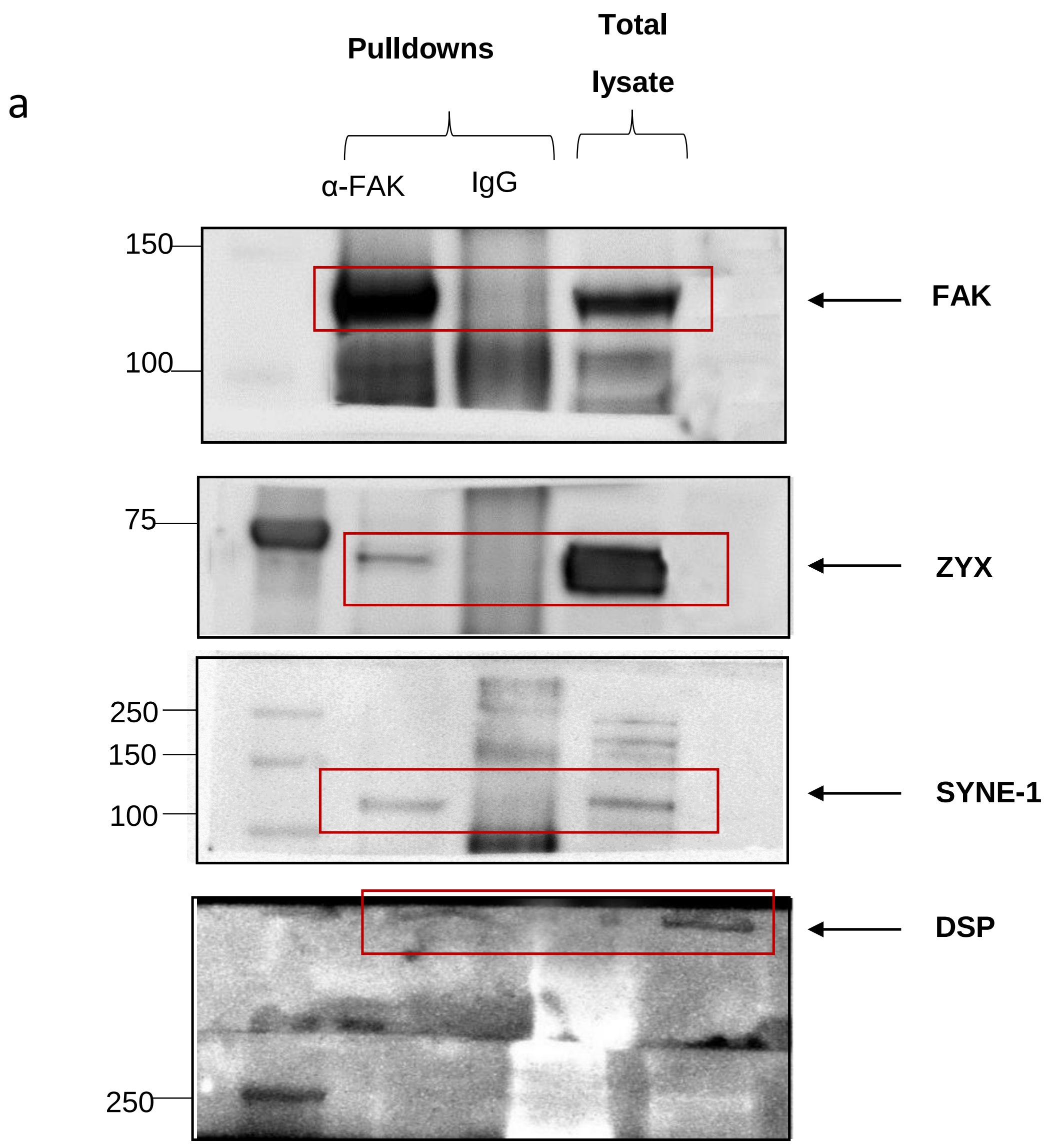

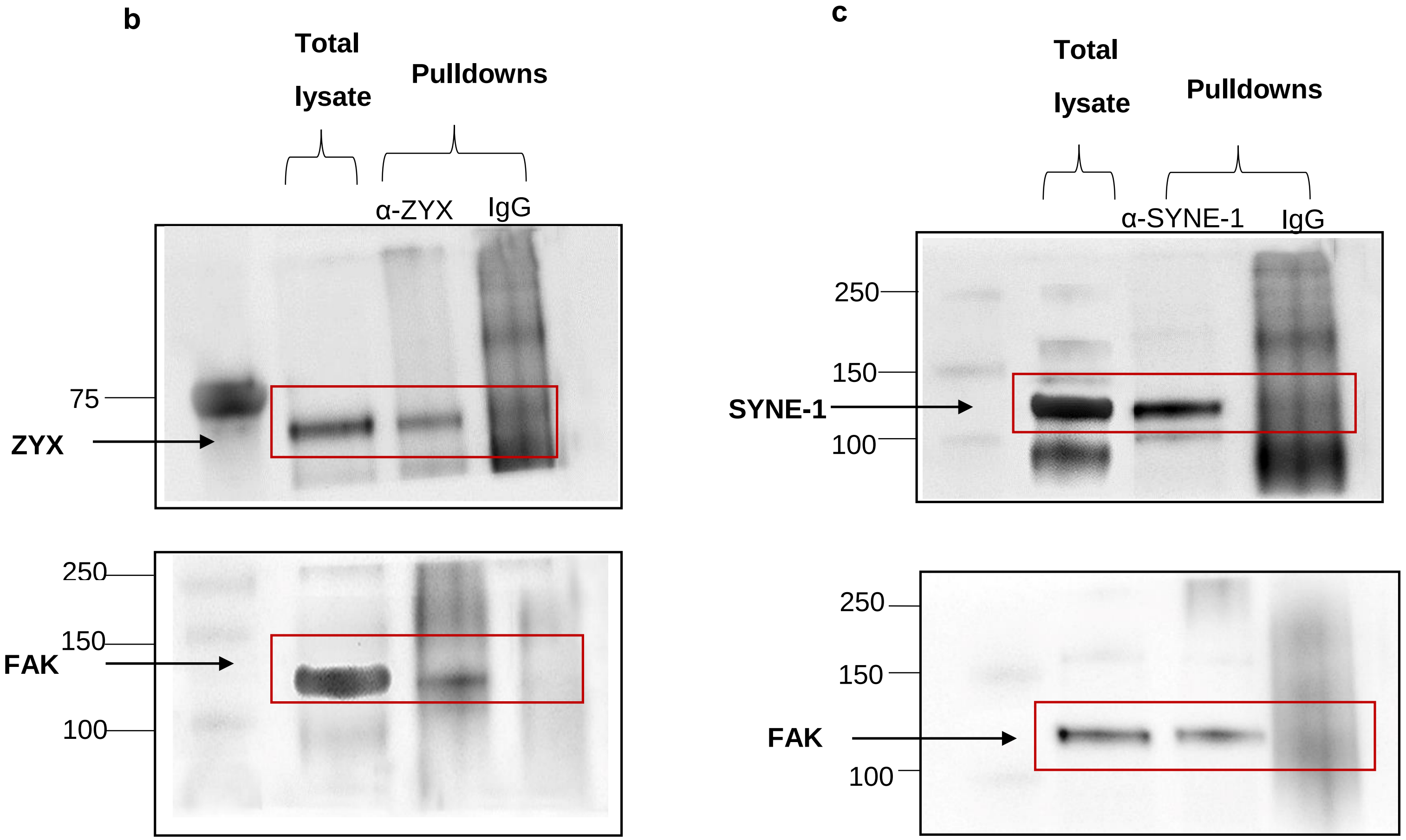
b
Total lysate
Pulldowns
α-ZYX
IgG
75
ZYX
250
150
FAK
100
c
Total lysate
Pulldowns
α-SYNE-1
IgG
250
150
SYNE-1
100
250
150
FAK
100

Fig. S6. Quiescent HCT-116 cells were transfected with siRNAs of zyxin, nesprin-1, and desmoplakin at different concentration of siRNAs. Densitometric quantification indicate the siRNA efficiency.

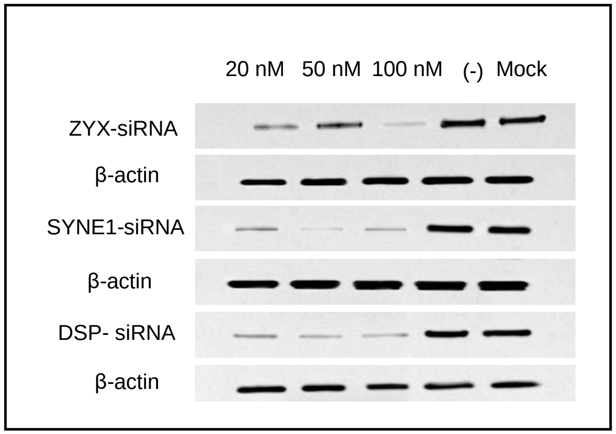

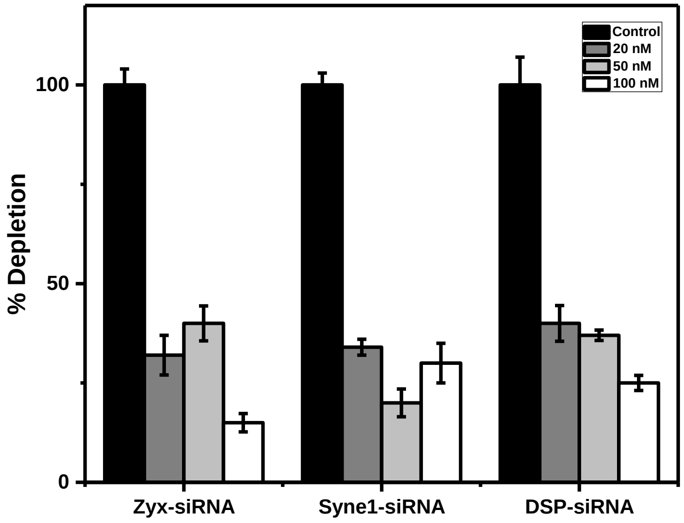

Fig. S7. Change in HCT-116 cell morphology after siRNA-transfection. a) untreated HCT-116 cells. b) ZYX-siRNA HCT-116 cells. c) SYNE-1-siRNA HCT-116 cells. d) DSP-siRNA HCT-116 cells. Images were capture after 24 h of transfection. HCT-116 cells lost their elongated shape to become oval shapes after treatment.

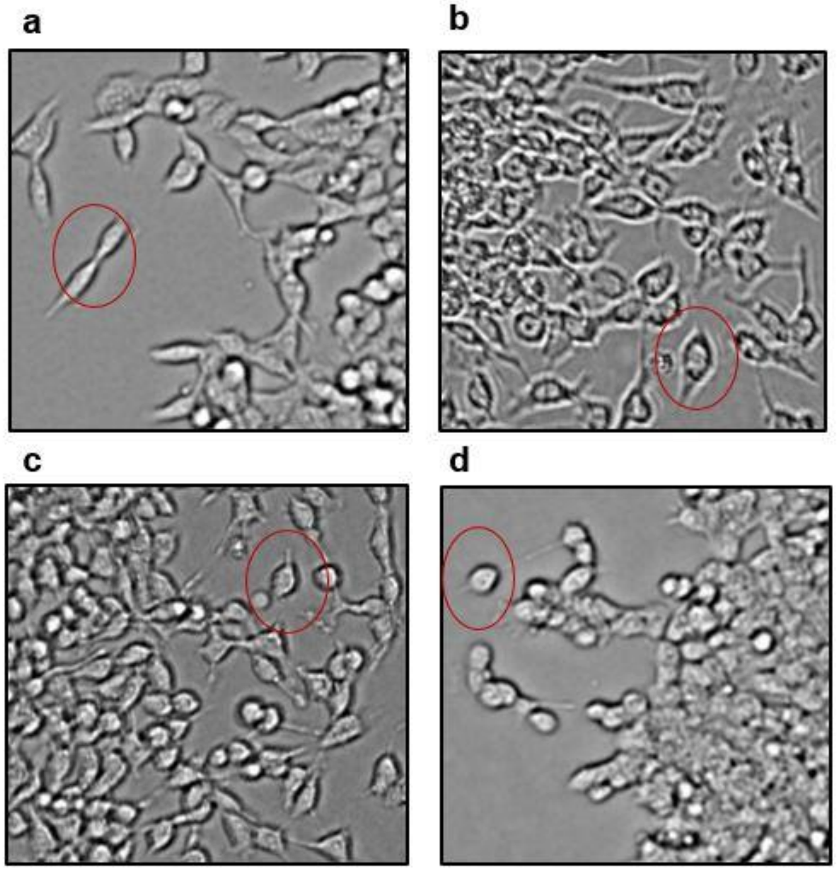

Fig. S8. Full blots of Figure 6 in the main text

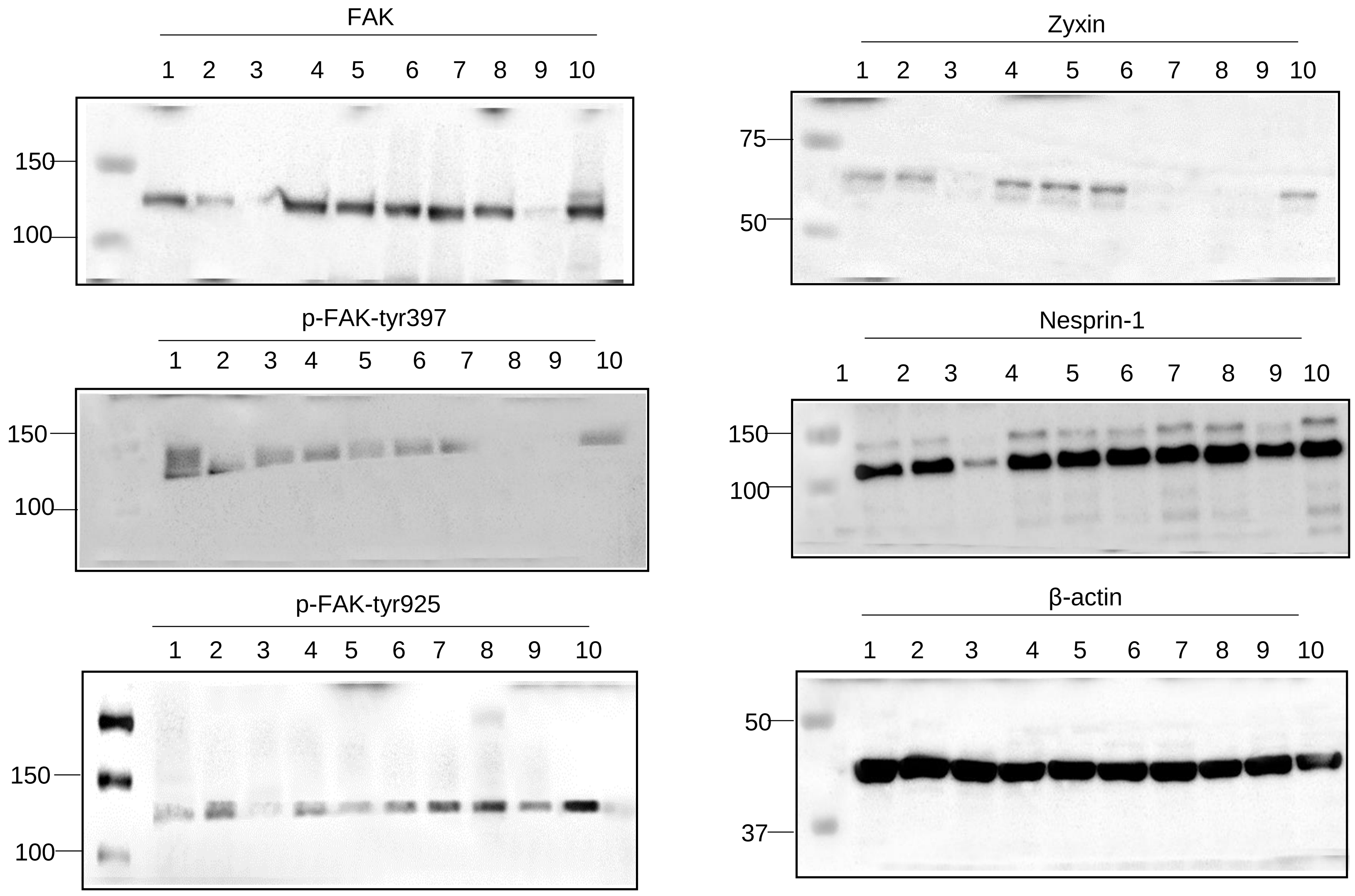

Fig.S9 Full blots of Figure 7 in the main text

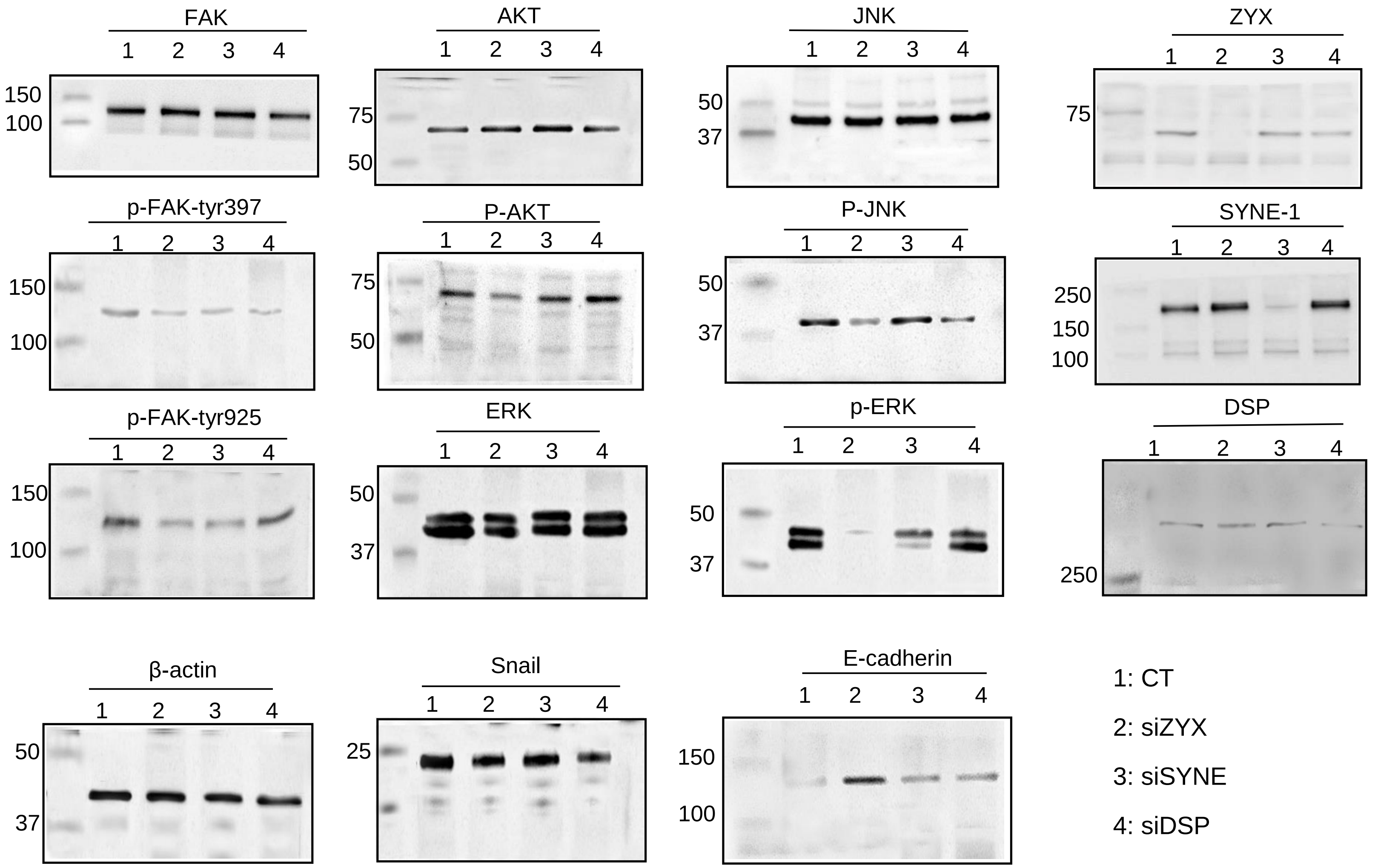

Fig S10. Full blot of Figure 9c in the main text

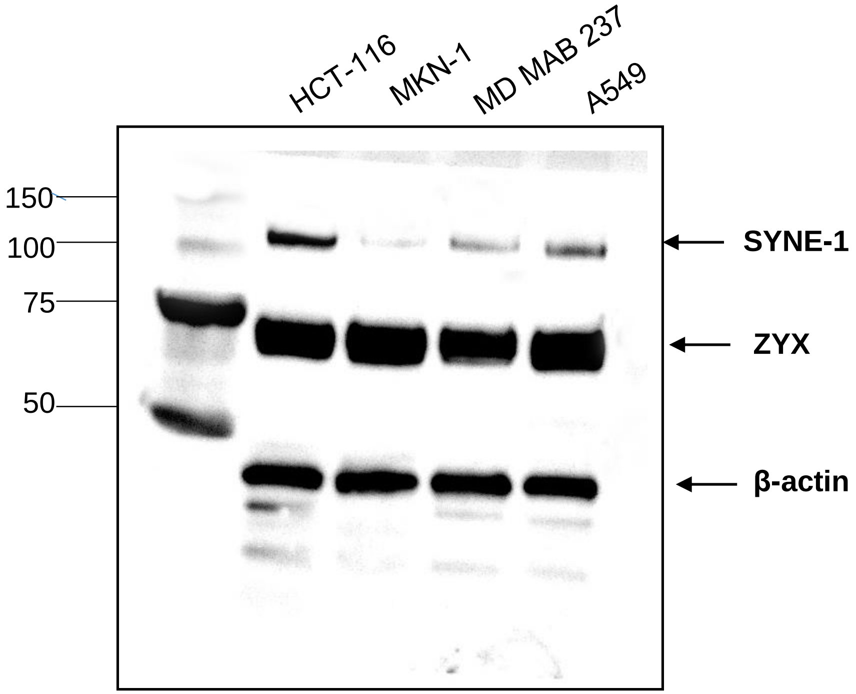

Fig. S11. **Ingenuity Pathway analysis (IPA) identified top significant network under EGF stimulation**. Major networks were identified after 30 min incubation with EGF and followed by FAK-IP. Top networks included differentially expressed genes**.** EGFR, PTK2, TP53, and CCND1 are central nodes of mechanistic network. Data were analyzed through the use of IPA (QIAGEN Inc., https://www.qiagenbioinformatics.com/products/ingenuitypathway-analysis

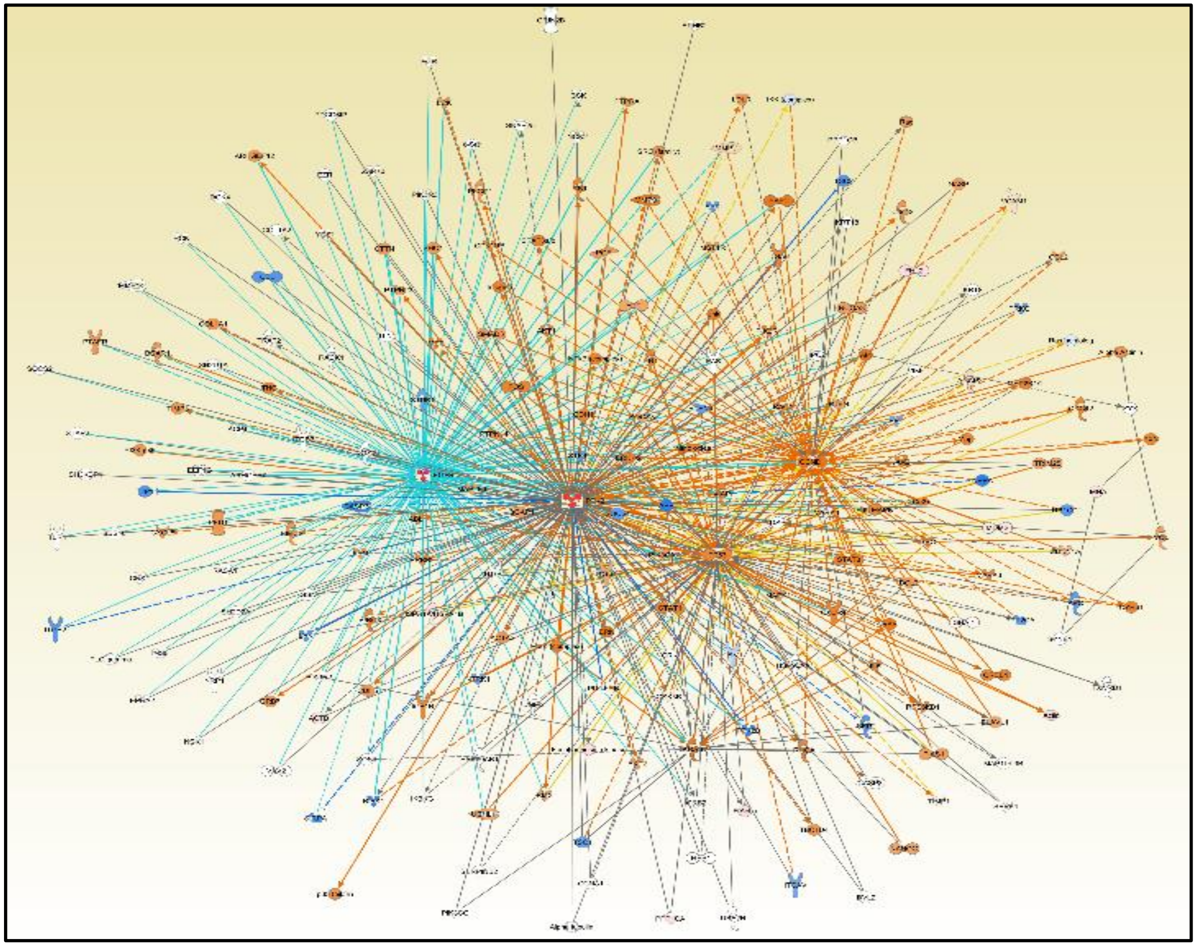

Table S1. Exclusive proteins identified from regular IP

Accession	Description	Xcorr (Top charge)	Score
**P51114-1**	Fragile X mental retardation syndrome-related protein 1	1.94 (+2)	38.5
**Q9UHB9**	Signal recognition particle subunit SRP68	2.03 (+2)	28.89
**O60506**	Heterogeneous nuclear ribonucleoprotein	2.41 (+2)	25.51
**Q15050**	Ribosome biogenesis regulatory protein homolog	1.92 (+1)	24.34
**P14136**	Glial fibrillary acidic protein	1.98 (+1)	21.03
**Q14444**	Isoform 2 of Caprin-1	2.84 (+2)	17.45
**O95782**	AP-2 complex subunit alpha-1	2.73 (+2)	16.32
**O76094**	Signal recognition particle subunit SRP72	3.55 (+3)	15.07
**Q5JTH9-1**	RRP12-like protein	3.71 (+3)	14.34
**Q99623**	Prohibitin-2	2.41 (+2)	13.01
**Q8IWS0-1**	PHD finger protein 6	3.35 (+3)	12.15
**Q8N0V3-1**	Putative ribosome-binding factor A, mitochondrial	3.43 (+3)	10.14
**Q14192**	Four and a half LIM domains protein 2	3.63 (+2)	10.81
**P07305**	Histone H1.0	3.61 (+2)	8.32
**P25391**	Laminin subunit alpha-1	2.67 (+1)	6.33

Table S2. De-novo sequencing of top peptides of FAK, zyxin, nesprin-1 and desmoplakin. Ions ascribed to the main fragmentation series, y (C-terminal series) and b (N-terminal series) are labeled. The amino acid sequence obtained by manual de novo sequencing is displayed showing assignment of y and b fragment ions of each peptides

				**FAK**				
**#1**	**b⁺**	**b²⁺**	**b³⁺**	**Seq.**	**y⁺**	**y²⁺**	**y³⁺**	**#2**
1	58.05872	29.53300	20.02442	G				21
2	205.23296	103.12012	69.08250	F	2508.80514	1254.90621	836.93990	20
3	318.39087	159.69907	106.80181	L	2361.63090	1181.31909	787.88182	19
4	432.49375	216.75051	144.83610	N	2248.47299	1124.74013	750.16252	18
5	560.62325	280.81527	187.54594	Q	2134.37011	1067.68869	712.12822	17
6	707.79749	354.40238	236.60401	F	2006.24061	1003.62394	669.41839	16
7	808.90157	404.95442	270.30538	T	1859.06637	930.03682	620.36031	15
8	938.01580	469.51154	313.34345	E	1757.96229	879.48478	586.65895	14
9	1053.10340	527.05534	351.70598	D	1628.84806	814.92767	543.62087	13
10	1181.27600	591.14164	394.43018	K	1513.76046	757.38387	505.25834	12
11	1278.39141	639.69934	426.80199	P	1385.58786	693.29757	462.53414	11
12	1379.49549	690.25138	460.50335	T	1288.47245	644.73986	430.16233	10
13	1492.65340	746.83034	498.22265	L	1187.36837	594.18782	396.46097	9
14	1606.75629	803.88178	536.25695	N	1074.21046	537.60887	358.74167	8
15	1753.93052	877.46890	585.31503	F	960.10757	480.55743	320.70738	7
16	1901.10476	951.05602	634.37311	F	812.93334	406.97031	271.64930	6
17	2064.27841	1032.64284	688.76432	Y	665.75910	333.38319	222.59122	5
18	2192.40791	1096.70759	731.47415	Q	502.58546	251.79637	168.20000	4
19	2320.53741	1160.77234	774.18399	Q	374.45595	187.73162	125.49017	3
20	2419.66870	1210.33799	807.22775	V	246.32645	123.66686	82.78034	2
21				K	147.19516	74.10122	49.73657	1
				**Zyxin**				
1	148.18151	74.59440		F				16
2	235.25898	118.13313		S	1369.41950	685.21339		15
3	332.37439	166.69083		P	1282.34204	641.67466		14
4	389.42583	195.21656		G	1185.22663	593.11695		13

5	460.50389	230.75558		A	1128.17519	564.59123		12
6	557.61931	279.31329		P	1057.09713	529.05220		11
7	614.67075	307.83901		G	959.98171	480.49449		10
8	671.72219	336.36473		G	902.93027	451.96877		9
9	758.79965	379.90347		S	845.87883	423.44305		8
10	815.85110	408.42919		G	758.80136	379.90432		7
11	902.92856	451.96792		S	701.74992	351.37860		6
12	1031.05806	516.03267		Q	614.67246	307.83987		5
13	1128.17347	564.59038		P	486.54296	243.77512		4
14	1242.27636	621.64182		N	389.42754	195.21741		3
	1370.40586	685.70657		Q	275.32466	138.16597		2
				K	147.19516	74.10122		1
				Nesprin-1				
1	100.07569	50.54148	34.03008	V				26
2	171.11280	86.06004	57.70912	A	2810.30051	1405.65389	937.43835	25
3	258.14483	129.57605	86.71980	S	2739.26339	1370.13533	913.75932	24
4	371.22890	186.11809	124.41448	L	2652.23136	1326.61932	884.74864	23
5	499.28747	250.14738	167.10068	Q	2539.14730	1270.07729	847.05395	22
6	614.31442	307.66085	205.44299	D	2411.08872	1206.04800	804.36776	21
7	745.35490	373.18109	249.12315	M	2296.06178	1148.53453	766.02544	20
8	832.38693	416.69710	278.13383	S	2165.02129	1083.01429	722.34528	19
9	992.41758	496.71243	331.47738	C-Carbamidomethyl	2077.98927	1039.49827	693.33461	18
10	1120.47616	560.74172	374.16357	Q	1917.95862	959.48295	639.99106	17
11	1233.56022	617.28375	411.85826	L	1789.90004	895.45366	597.30486	16
12	1346.64428	673.82578	449.55295	L	1676.81598	838.91163	559.61018	15
13	1445.71270	723.35999	482.57575	V	1563.73191	782.36959	521.91549	14
14	1559.75562	780.38145	520.59006	N	1464.66350	732.83539	488.89268	13
15	1630.79274	815.90001	544.26910	A	1350.62057	675.81392	450.87837	12
16	1759.83533	880.42130	587.28329	E	1279.58346	640.29537	427.19934	11
17	1816.85680	908.93204	606.29045	G	1150.54086	575.77407	384.18514	10
18	1917.90447	959.45588	639.97301	T	1093.51940	547.26334	365.17798	9
19	2032.93142	1016.96935	678.31532	D	992.47172	496.73950	331.49543	8
20	2192.96207	1096.98467	731.65887	C-Carbamidomethyl	877.44478	439.22603	293.15311	7
21	2306.04613	1153.52670	769.35356	L	717.41413	359.21070	239.80956	6
22	2435.08872	1218.04800	812.36776	E	604.33007	302.66867	202.11487	5
23	2506.12584	1253.56656	836.04680	A	475.28747	238.14738	159.10068	4
24	2634.22080	1317.61404	878.74512	K	404.25036	202.62882	135.42164	3

25	2763.26339	1382.13533	921.75932	E	276.15540	138.58134	92.72332	2
26				K	147.11280	74.06004	49.70912	1

Desmoplakin

#1	88.03930	44.52329		S				15
1	159.07642	80.04185		A	1754.88471	877.94599		14
2	272.16048	136.58388		I	1683.84759	842.42744		13
3	435.22381	218.11554		Y	1570.76353	785.88540		12
4	563.28239	282.14483		Q	1407.70020	704.35374		11
5	676.36645	338.68686		L	1279.64162	640.32445		10
6	805.40905	403.20816		E	1166.55756	583.78242		9
7	934.45164	467.72946		E	1037.51497	519.26112		8
8	1063.49423	532.25075		E	908.47237	454.73983		7
9	1226.55756	613.78242		Y	779.42978	390.21853		6
10	1355.60015	678.30371		E	616.36645	308.68686		5
11	1469.64308	735.32518		N	487.32386	244.16557		4
12	1582.72714	791.86721		L	373.28093	187.14410		3
13	1695.81121	848.40924		L	260.19687	130.60207		2
14				K	147.11280	74.06004		1
15								
